# The genome of *Eimeria falciformis* - reduction and specialization in a single host apicomplexan parasite

**DOI:** 10.1186/1471-2164-15-696

**Published:** 2014-08-20

**Authors:** Emanuel Heitlinger, Simone Spork, Richard Lucius, Christoph Dieterich

**Affiliations:** Department of Molecular Parasitology, Humboldt University, Philippstraße 13, 10115 Berlin, Germany; Computational RNA Biology and Ageing, Max Plank Institute for Biology of Ageing, Joseph-Stelzmann Straße 9b, 50913 Cologne, Germany

## Abstract

**Background:**

The phylum Apicomplexa comprises important unicellular human parasites such as *Toxoplasma* and *Plasmodium. Eimeria* is the largest and most diverse genus of apicomplexan parasites and some species of the genus are the causative agent of coccidiosis, a disease economically devastating in poultry. We report a complete genome sequence of the mouse parasite *Eimeria falciformis*. We assembled and annotated the genome sequence to study host-parasite interactions in this understudied genus in a model organism host.

**Results:**

The genome of *E. falciformis* is 44 Mb in size and contains 5,879 predicted protein coding genes. Comparative analysis of *E. falciformis* with *Toxoplasma gondii* shows an emergence and diversification of gene families associated with motility and invasion mainly at the level of the Coccidia. Many rhoptry kinases, among them important virulence factors in *T. gondii*, are absent from the *E. falciformis* genome. Surface antigens are divergent between Eimeria species. Comparisons with *T. gondii* showed differences between genes involved in metabolism, N-glycan and GPI-anchor synthesis. *E. falciformis* possesses a reduced set of transmembrane transporters and we suggest an altered mode of iron uptake in the genus *Eimeria*.

**Conclusions:**

Reduced diversity of genes required for host-parasite interaction and transmembrane transport allow hypotheses on host adaptation and specialization of a single host parasite. The *E. falciformis* genome sequence sheds light on the evolution of the Coccidia and helps to identify determinants of host-parasite interaction critical for drug and vaccine development.

**Electronic supplementary material:**

The online version of this article (doi:10.1186/1471-2164-15-696) contains supplementary material, which is available to authorized users.

## Background

The taxon *Eimeria* is the largest genus of the phylum Apicomplexa with more than 1,800 species [1]. The Apicomplexa are obligate intracellular parasites and the phylum contains many well-known pathogens of humans and livestock. *Plasmodium* species causing malaria can be regarded as the most threatening eukaryotes to human [2]. The causative agent of toxoplasmosis, *Toxoplasma gondii*, infects about a third of the worldwide human population and causes pathology mostly in immunodeficient individuals and neonates [3].

Eimeriids dwell within cells of the intestine and intestine-related tissues of vertebrates and invertebrates. The family comprises human pathogenic parasites like *Isospora belli* and *Cyclospora caytanensis*. The genus *Eimeria* does not infect humans, but is best known for species infecting domestic animals. *Eimeria* species cause > 2 billion US$ damage p. a. alone in the poultry industry [4]. Eimeriid parasites have single host life cycles and typically display a strict host and tissue specificity. For example, the chicken is parasitized by 7 species of Eimeria, each restricted to a special part of the intestine and causing severe, self-limiting infections [4].

**Figure 1:**
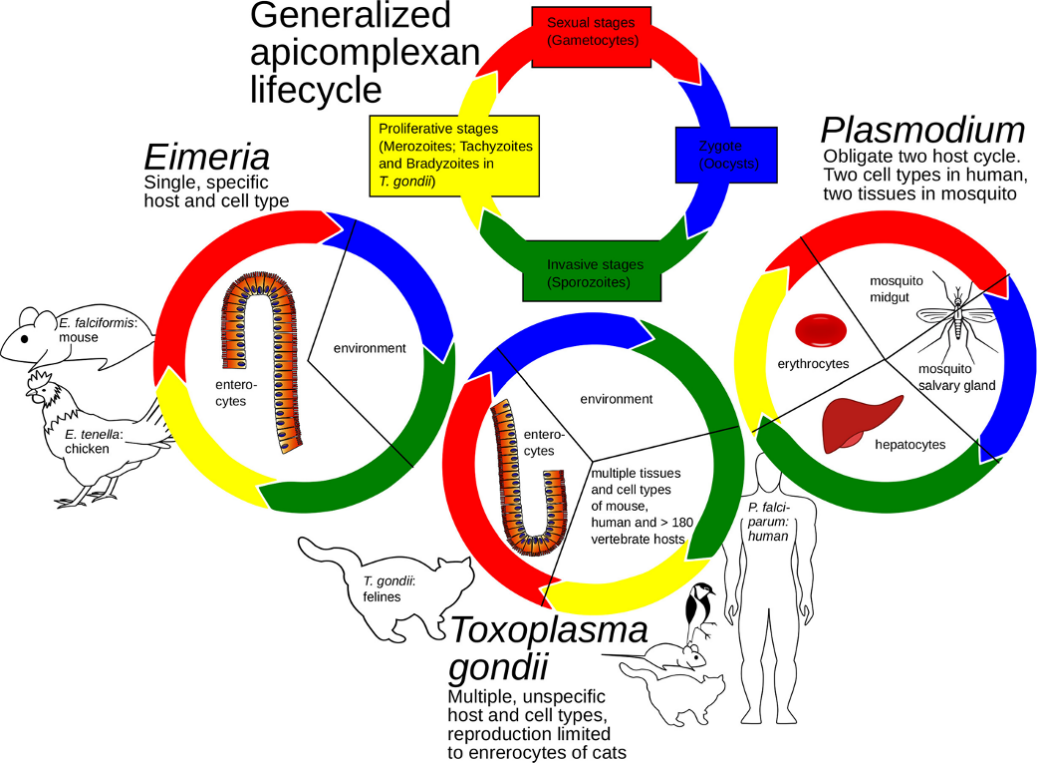
**Lifecycle of** ***Eimeria*** **compared to other Apicomplexans.** The lifecycles of apicomplexan parasites are illustrated to highlight the differences in host and cell type specificity. *Eimeria* parasites have a single host lifecycle and show a strict tissue and cell type specificity. *T. gondii* can infect multiple hosts and different tissues and cell types. Sexual reproduction, however, is restricted to epithelial cells of cats. *Plasmodium falciparum* has an obligate two host lifecycle, proliferative stages are only found in humans, sexual reproduction takes place in mosquitoes.

The development of E. *falciformis* Eimer, 1870 [5] is restricted to crypt epithelial cells of the cecum and proximal colon of the mouse [6, 7]. *E. falciformis* combines the typical life cycle elements of parasites of the subclass Coccidia (Figure 1): Infection occurs by ingestion of oocysts, containing eight haploid sporozoites. Sporozoites infect epithelial cells and undergo several rounds of asexual replication (“schizogony”), leading to very high numbers of progeny called merozoites. These differentiate into gametes (“gamogony”) that fuse to form diploid zygotes. After leaving the host via the feces in environmentally resistant oocysts they undergo a reduction division and mitotic divisions (“sporogony”) to yield the infective sporozoites. As its host, the mouse, is among the best-studied systems in biological research, the *E. falciformis* infection lends itself as a model to dissect facets of *Eimeria*-host interaction [8–11].

*Plasmodium* is a Haemosporidian only distantly related to Eimeria and the Coccidia. *T. gondii*, the only member of the genus *Toxoplasma,* belongs to the same subclass as the Eimeriidae, the Coccidia. Its life cycle shows similarities to that of Eimeria in its feline definitive hosts, but in contrast to the specialist, it can infect >180 species of warm blooded animals as intermediate hosts, including humans [3]. Due to this flexibility, this relatively easy to handle parasite has become a well-studied model organism that has greatly furthered the understanding of apicomplexan biology. It has been argued that *T. gondii* needs its relatively large genome to adapt to its wide variety of intermediate hosts, requiring for example flexibility with regard to host metabolism or immune reposes. Interestingly, *T. gondii* has diverged into strains with different pathogenicity, supposedly reflecting adaptation to the immune responses of its intermediate hosts [12]. The factors, which led to the divergence of Eimeriids into specialist species and to the coherence of *T. gondii* as one generalist species, are still not understood.

Previously, high-quality genome sequences have been reported for *T. gondii*[13] and its very close relatives in the family Sarcocystidae *Neospora caninum*[14] and *Hammondia hammondii*[15]. In the family Eimeriidae, first studies of *E. tenella* chromosome I [16] and the description of a database for the genome assembly of *Eimeria maxima*[17] were complemented by a large scale investigation of all 7 species of *Eimeria* infecting chickens recently [18]. Genome data has not been reported for any *Eimeria* species infecting a host other than chicken.

We determined a draft genome sequence of the mouse parasite *E. falciformis* using a next generation sequencing approach, to promote studies on the biology of Eimeriids. We performed comparative genomic analyses and focused on the congener *E. tenell*a and two more distant relatives, *T. gondii and N. caninum,* to infer the evolutionary history of genes playing roles in core processes in these parasites.

One such core process, the substrate dependent locomotion of coccidian and haemosporidian parasites is called gliding motility. An actin-dependent motor drives this process [19]. This movement is the basis for the invasion process, during which invasive stages of apicomplexan and especially coccidian parasites establish themselves in a parasitophorous vacuole. The secretion of surface molecules from specialized apical organelles called micronemes is triggered by cyclic nucleotide and calcium signalling and transduced by calcium dependent kinases. Other apical organelles, the rhoptries secrete kinases (Rhoptry kinases; RopKs) and pseudokinases interacting with the host immune system [20].

Comparative genomics of the complete *E. falciformis* genome allow a comprehensive view of the shared gene repertoire among apicomplexan and coccidian parasites. Our data for a rodent Eimeriid species allows identifying genus specific processes and molecules. The presented genome sequence thus provides a better understanding of the biology of Eimeriids and a basis to establish *E. falciformis* as a suitable model-parasite.

## Results and discussion

### Genome sequence, size and completeness

We determined the draft genome sequence of the mouse parasite *Eimeria falciformis* using a next generation sequencing approach comprising one standard paired-end and one mate pair library with ~ 2 Kilobase (Kb) insert size sequenced with Illumina technology. A total nominal sequencing coverage of more than 500× allowed the construction of a largely contiguous whole genome sequence. We assembled 753 high quality contigs covering a sequence space of 43.7 Mb with a median weighted contig size (N50) of ~250 kb and thus conclude a genome size of roughly 44 Mb. The five longest contigs of the assembly cover a combined length of 4.6 Mb and could very well represent some of the typically 14 chromosomes found in *Eimeria* species and other Coccidia [21]. With this size the genome of *E. falciformis* is smaller than those of other *Eimeria* species [18]. It ranges between the size of the strongly reduced genomes of *Cryptosporidium parvum* and the relatively large one of *T. gondii* (Table 1)*.* We have submitted the *E. falciformis* data to the Short Read Archive as project SRP034650, the genome assembly and relevant annotations will also be accessible through ToxoDB [13].

**Table 1:** **Genome features of model apicomplexa**

	Genome size	# genes	Median cds exons/gene	Median gene-length	Median transcript-length	Median intron length
*Cryptosporidium hominis*	8,743,570	3,956	1	1,034	1,044	54
*Cryptosporidium parvum*	9,102,324	3,886	1	1,280	1,302	61
*Eimeria falciformis*	43,683,212	5,879	6	3,300	1,584	183
*Eimeria tenella*	51,807,230	9,262	3	1,593	1,032	222
*Neospora caninum*	61,040,345	7,227	5	3,544	1,656	433
*Babesia bovis*	8,179,706	3,781	2	1,296	1,143	36
*Theilieria annulata*	8,358,425	3,845	3	1,406	1,227	48
*Plasmodium berghei*	18,560,668	5,127	2	1,572	1,014	106
*Plasmodium falciparum*	23,332,831	5,772	2	1,620	1,360	141
*Toxoplasma gondii*	63,005,196	8,155	4	3,594	1,464	477

### Organellar genomes

Most apicomplexan parasites harbor a mitochondrion and a rudimentary plastid organelle, the apicoplast, both of which contain extra-nuclear DNA. We estimate the size of both organellar genomes to 6.2 kb and ~33 kb, respectively. Both genomes are present in higher copy numbers relative to the nuclear genome as estimated from sequencing coverage (see Additional file 1). We predict ~180 copies per cell for the mitochondrial genome and 18–19 copies per cell for the apicoplast genome.

The mitochondrial genome of *E. falciformis* is in good agreement with GC content (34.5%) and size (6.2 kb) estimates for mitochondrial genomes of avian *Eimeria* species [22]. For the protein encoding genes of the mitochondrial genome cytochrome c oxidase subunits 1 (cox1), 3 (cox3) and cytochrome oxidase b (cytb) expression evidence was found in RNA-Seq data.

Our attempts to assemble a single contiguous apicoplast genome sequence did not succeed with the available data. By comparison to the *E. tenella* plastid genome [23] we inferred the repeat structure of the ribosomal genes (two inverted repeats) as the likely cause of the assembly problems. Hence we reconstructed the *E. falciformis* apicoplast genome by assuming conserved synteny with the *E. tenella* genome (Additional file 2). The 33 kb apicoplast genome is AT rich with a GC content of just ~23%. Apicoplast encoded genes were not represented in our RNA-Seq data. It remains to be tested whether this is due to a lack of transcript polyadenylation in this organelle. In dinoflagellates polyadenylation has been used as an indication for nuclear origin (instead of plastid origin) before [24].

### Genome structure

The overall GC content of the nuclear genome (52.9%) is similar to the *E. tenella* genome (51.3%). We found trimer-repeats of CAG/GTC (repeated 9–16 times) and heptamer-repeats of AAACCCT/AGGGTTT (repeated 6–14 times) as the most abundant simple sequence repeat (SSRs) instances. Another considerable proportion of the genome (9.6 Mb; 22%) consists of more complex non-SSR elements. Both SSR and complex repeats are spread in a pattern consistent with the segmental organization of the *E. tenella* and other chicken Eimeria genomes [16, 18], i.e. they occur together and are not confined to few telomeric regions.

Trimer repeats (and other k-mer repeats, where k is a multiple of 3) are often (58% of cases) found in protein coding sequences, heptamer and other “frame-incompatible” repeats are found outside of coding sequence in 93% of their occurrences.

The specific base combination CAG (repeated at least 7 times) is found in 1815 genes, leading to conceptual translations into homopolymeric amino acid repeats (HAARs) of alanine (A), glutamine (Q) or serine (S) in 1781 (30%) proteins. This is a smaller proportion compared to the 57% of genes containing HAARs in *E. tenella*[18]. Nevertheless the identical conservation of nucleotide sequence coding for HAARs but also non-coding heptamer repeats outside of protein coding genes is remarkable. It suggests a non-protein-mediated functional role e.g. in nuclear organization or as a second possibility the conservation of the generating mechanism and a near neutrality of the SSRs and the resulting HAARs.

The nuclear genome contains 151 functional tRNA genes for 46 codons of 20 standard proteinogenic amino acids. These tRNAs genes were found on 84 different contigs, with no contig containing more than 10 tRNAs genes. Clusters of multiple rRNA genes for the nuclear large and small ribosomal subunits (LSU and SSU) were found on 3 contigs. Additional single copies of LSU and SSU were found on 6 and 2 contigs, respectively. SSU displayed polymorphism (both SNPs and indels) in alignable regions of different rRNA genes.

Combining expression evidence from RNA-seq data with *ab initio* predictions we inferred 5,879 protein-coding genes producing 6,586 transcripts and predicted conceptual translations for proteins. We estimate that 28.2 Mb (~64.5%) of the *E. falciformis* genome is contained within protein coding gene loci with 14.6 Mb (~33%) coding sequence. Coding sequence exons typically show a higher GC (55.9%) content than introns (47.1%) (Additional file 3).

Protein coding genes of *E. falciformis* have a median span of 3.3 kb (Table 1). As a result of both a higher number per gene and longer introns (Additional file 4) Coccidia have longer genes then e.g. the Haemosporidia. The genomes of *Plasmodium* species contain mainly small sized exons, but a distinct population of larger exons is also found. These are comparable in size to the single exon genes found in *Cryptosporidium* species. Partial or complete intron loss, in these genomes, resulting in merged exons has been ascribed to bursts of transposon activity [25]. No similar signs of intron loss as relics of past or present transposon activity are found in protein coding genes of *E. falciformis.*

The location of regions with similarity to protein coding genes of transposons (TransposonPSI), however, was found to correlate with repetitive regions. This might indicate a role of these (retro-)transposon elements in the acquisition and spread of repeats. *W*e thus suggest that transposons have not had a large role in shaping the protein coding genes of Coccidia and especially *E. falciformis* but potentially had a role in the emergence of the non-coding repeat repertoire. Detailed organization, categorization and origin of these repeats constitutes an interesting area of research for comparative genomics within the genus *Eimeria* as more complete genome sequences become available.

**Figure 2:**
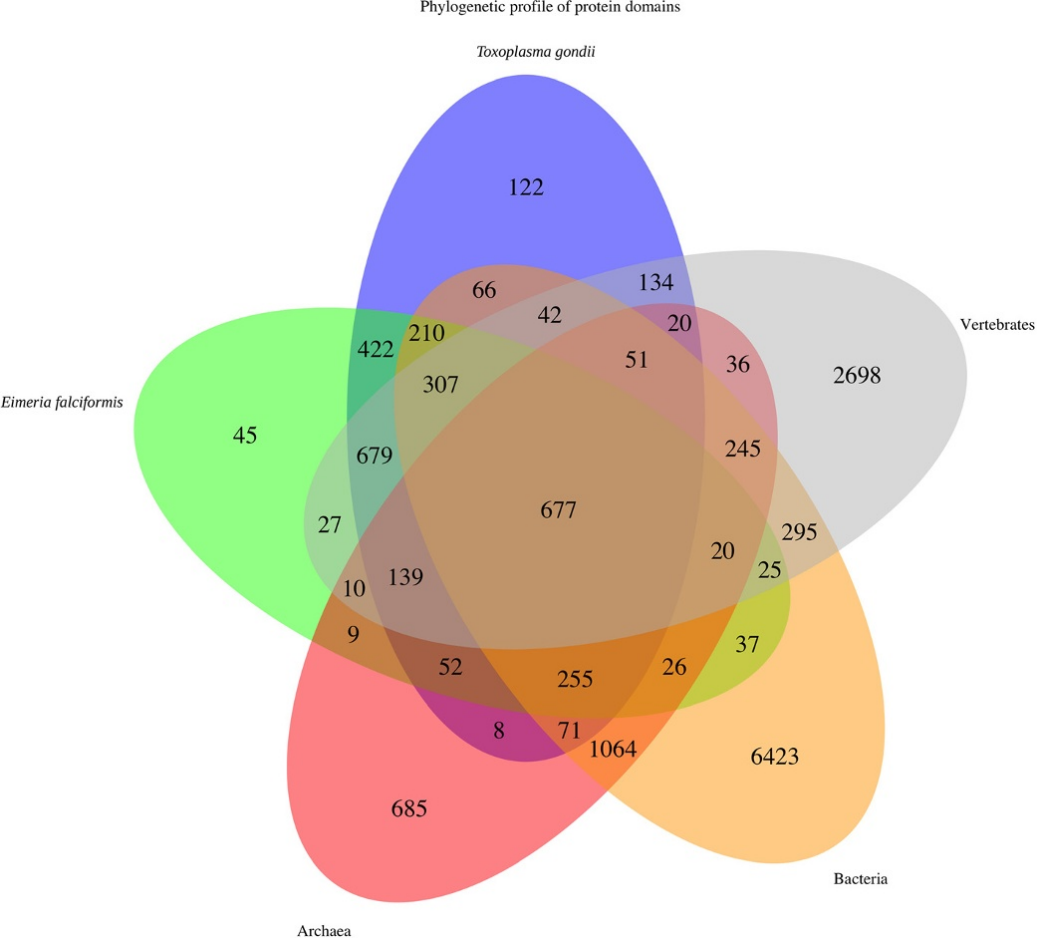
**Phylogenetic profile of protein domains across** ***E. falciformis*** **,** ***T. gondii*** **and three phyla.** We annotated protein domains for the *E. falciformis* and *T. gondii* genome with InterproScan (v5 RC4) [26]. Complete protein InterPro domain annotations for vertebrates, archaea and bacteria were obtained from uniprot.org. Protein domains that were exclusively annotated in *E. falciformis* (45 domains), *E. falciformis* + vertebrates (27 domains) and *E. falciformis* but not in *T. gondii* (45 + 27 + 10 + 9 + 20 + 26 + 37 + 25) show an enrichment for specific GO terms (see main text).

As observed by Reid et al. [18] repeats are not found within the functional domains of protein coding genes. We obtained domain annotations using InterProScan [26] for 3,587 (54.4%) of our predicted proteins and found 23,059 domain occurrences, representing 2,940 distinct InterPro accessions (Additional file 5). Based on these annotations Gene Ontology (GO) [27] terms could be obtained for 2,841 genes (Additional file 6). For a direct comparison of proteomes, we also predicted 26,584 domains in 4,498 genes covering 3,321 distinct accessions in *T. gondii* (Additional file 7). Comparison of the Interpro domain annotations of *E. falciformis* with those obtained for the *T. gondii* proteome and with protein domain annotations from all vertebrate, bacterial and archaeal proteomes as listed in SwissProt (Figure 2) yielded species-specific sets of domains.

### Comparative functional analyses of coccidian proteomes

It is conceivable that the diversity of encoded proteins should vary with the number and complexity of the niches a pathogen meets in its host(s). If this held true, *Eimeria* parasites dwelling within relatively specialized cells like enterocytes or endothelial cells of a single host can be expected to have a less complex protein repertoire, as compared to parasites exploiting several very different niches in two hosts, like *T. gondii*. To elaborate specific biological functions of Eimeriids, we clustered 118,629 proteins from apicomplexan genomes plus proteins from *Arabidopsis thaliana*[28], *Saccharomyces cerevisiae*[29] and the diatom *Thalassiosira pseudonana*[30] as outgroup. We obtained 76,740 ortholog clusters, containing 4,466 *E. falciformi*s genes in 4,205 clusters of which 4,186 contained additional genes from other species (Table 2). This analysis also demonstrates that our *E. falciformis* gene-set is complete for conserved genes of apicomplexan parasites. A comparison of the size of gene sets of *E. falciformis* and *E. tenella* (5,879 vs. 9,262 predicted genes) hence suggests that the smaller gene-set might be less redundant.

**Table 2:** **Summary of ortholog clusters**

	# clusters	# clustered genes	# pan apicomplexan missing	# paralog only clusters	# paralog only genes
*Eimeria falciformis*	4205	4466	98	19	62
*Eimeria maxima*	2431	3975	NA	298	934
*Eimeria tenella*	4226	5020	157	61	244
*Toxoplasma gondii*	6155	6581	7	24	67
*Neospora caninum*	6075	6572	11	11	45
*Babsia bovis*	2726	3164	32	34	362
*Theilieria annulata*	2655	3170	64	37	281
*Plasmodium falciparum*	3924	4617	5	51	568
*Plasmodium berghei*	3893	4253	3	18	262
*Cryptosporidium hominis*	1751	1885	NA	33	88
*Theillasiodosira pseudonana*	3808	6638	NA	784	2872
*Arabidopsis thaliana*	5834	22732	NA	2574	13583
*Saccharomyces cervesiae*	2433	3667	NA	325	1032

**Figure 3:**
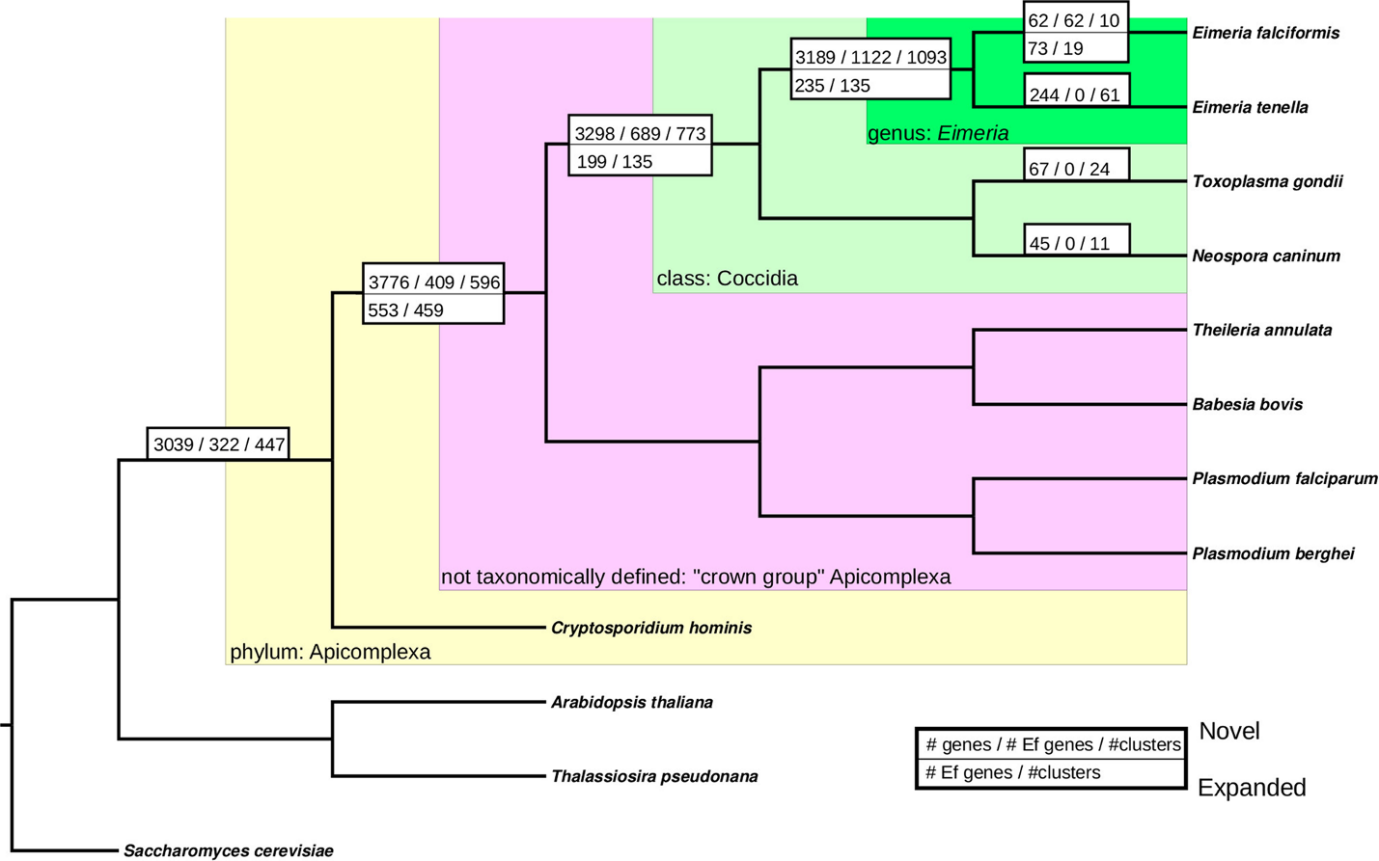
**Classification of novel and expanded ortholog clusters based on phylogenetic profiles.** Ortholog clusters were predicted using OrthoMCL [32] and species phylogeny was constructed from a matrix of presence and absence of ortholog clusters using maximum parsimony. The clade given as “crown group apicomplexa” is taxonomically not defined but comprises the Apicomplexa under exclusion of *Cryptosporidium*. Numbers in the upper half of the boxes present the total number of genes, of *E. falciformis* genes and of ortholog clusters novel in a given clade. Numbers are not given cumulative, and the listed lower level genes/clusters are not contained within the higher levels. Novelty was inferred from the taxonomic range of constitutive member genes for each cluster. The lower half of boxes gives numbers for *E. falciformis* genes and ortholog clusters expanded at a given node. Expansion was inferred from reconciliation of individual gene trees (see Methods) with the species tree. Gene families with at least one gene duplication event at a given node were considered expanded. We limited these analyses to the branches leading to *E. falciformis*.

To obtain information on the occurrence of biological specializations, we assigned ortholog clusters into categories based on their phylogenetic profile (Figure 3). Our analysis identified a high number of genus *Eimeria* and Coccidia-specific gene clusters, which is in agreement with previous findings [31]. These novel or diverged gene-families are expected to contain many of the genes responsible for the diverse biological specializations related to differences in modes of parasitism (e.g. host-usage) and life cycle (incl. reproduction). We thus profiled the clusters extensively for enrichment of particular functions (Additional file 8).

To further infer the evolutionary history of these gene clusters, we constructed phylogenetic trees for all ortholog clusters and compared these with the species phylogeny and identified gene families that underwent noticeable expansion events. We detected 22 gene clusters expanded at the species level in *E. falciformis*. In 135 ortholog clusters expansions at the level of the genus *Eimeria* was inferred (with 235 *E. falciformis* genes). Moreover, 135 ortholog clusters (199 *E. falciformis* genes) showed Coccidian specific expansions and 456 ortholog clusters (553 *E. falciformis* genes) expanded at the “crown group Apicomplexa” level. Within the latter ortholog clusters, copies of *E. falciformis* genes were obviously often lost. Additional file 9 highlights functions overabundant in clusters expanded in particular clades of the Apicomplexa.

Some ortholog clusters showed continued expansion throughout their evolutionary history: A cluster of myosin head domain containing proteins for example started expanding early in the evolution of the Apicomplexa and continued to expand to the genus level in *Eimeria*. This occurrence of a large cluster of genes with motor activity and myosin heads expanding throughout the phylogeny is accompanied by diversification into novel or highly diverged ortholog clusters with the same annotation especially at the Coccidia level. This can be interpreted as a continued evolutionary invention of slightly altered functions for these motility associated genes.

The phylogenetic distance between *E. falciformis* paralogs in ortholog clusters expanded at the class or genus level is larger compared to the most recent expansions at the species level. These most recent *E. falciformis* paralogs are likely less diverged simply due to a shorter time allowed for divergence. More intriguingly paralogs with a (sub-)class or genus origin have also a higher average distance than those arising basally in the Apicomplexa (Additional file 10). The function of these older paralogs gives a hint on possible reasons. Genes in clusters expanded basally in the Apicomplexa are involved in conserved processes like transcription, translation and metabolism. The acquisition of related core functions of novel paralogs could explain their low divergence. In contrast neofunctionalization of novel paralogs during the cladogenesis of the Coccidia and the genus *Eimeria,* accompanied by the acquisition of new niches, could have produced more divergent proteins.

#### Interdependent gene family expansions in Eimeriidae and Sarcocystidae

Reconciliation of gene trees with the species tree allowed us to highlight cases of independent gene family expansions in both branches of the Coccidia, namely Eimeriidae and Sarcocystidae. It can be hypothesized that in these cases functional niches similar in the Sarcocystidae and Eimeriidae could be filled by the new paralogs. These novel functions would in turn be highly interesting because of their repeated evolutionary invention.

We noticed such independently expanded gene families in total in 16 ortholog clusters, containing 26 *E. falciformis* genes. In ten of these families the duplicated paralog was only present in *E. tenella* and the remaining six clusters comprised 16 *E. falciformis* genes.

Two *E. falciformis* paralogs in one of these clusters were annotated as fructose-bisphosphate aldolase (FBA; Additional file 11). FBA typically functions as a metabolic enzyme in glycolysis and energy production. However, one of the *T. gondii* FBA paralogs is also essential for host cell invasion linking a micronemal protein to the cytoskeleton. This mechanism potentially allows the spatially focused supply of energy during invasion [33]. The independently arisen expansion of *Eimeria* paralogs poses the question whether Eimeria has developed new functions relating glycolysis to cell invasion in a parallel evolutionary process.

As a second illustrative example, membrane alanyl aminopeptidase showed independent expansions into multiple paralogs in *Eimeria* and Sarcocystidae (Additional file 12). In this case the complete set of paralog copies was only retained in the genomes of *E. falciformis* and *T. gondii* (3 and 4 respectively). In *Plasmodium,* the single orthologous protein is involved in hemoglobin digestion and is a possible drug target due to its indispensability to the parasite [34]. The function of these peptidases and their independent paralogous expansions and retention (and hence assumed neofunctionalisation) in some Coccidia awaits further investigation.

#### Surface antigens

Surface proteins are of particular interest for studies on pathogen host interactions as they are exposed on the outer membranes of parasites and are thus potential targets of protective immune reactions. Ortholog clusters with a phylogenetic distribution limited to the genus *Eimeria* were enriched for traits characteristic of surface proteins, namely sequences with secretion signal (SignalP) likely to be exported by the secretory pathway. Such signal-peptide carrying *E. falciformis* proteins were enriched for “serine-type endopeptidase activity” (GO:0004252). Coccidia-specific clusters on the other hand were enriched for sequences with other traits of surface proteins: signal anchors (SignalP) and transmembrane helices (TMHMM). Such transmembrane helices were also overrepresented in *E. falciformis* species specific clusters.

The domain “Pollen allergen Poa pIX/Phl pV” (IPR001778; found in seven genes) was found restricted to *E. falciformis*. The same domains are found in *Trypanosoma cruzi* mucin associated surface proteins (MASPs). MASPs are important antigenic peptides and coordinated expression of the MASPs repertoire is likely important for the evasion of host immune responses [35].

Our analysis also revealed 514 domains only present in *T. gondii* genes*,* but not in the *E. falciformis* gene set. The most prominent example among these was the SRS (SAG related; IPR007226) domain in 107 *T. gondii* genes. The SRS proteins are surface proteins putatively involved in binding to host cell receptors and attachment of the asexual replicative forms present in intermediate hosts [36].

By overlaying our protein domain annotation and OrthoMCL clustering results, we identified two surface antigen (SAG) clusters containing “Sporozoite TA4 surface antigen” domains in large paralog clusters of *E. falciformis* and *E. tenella*. The eponymic protein SAG1 induces antibody production in chickens and has been characterized and cloned from *E. tenella* sporozoites as a prominent vaccine candidate [37]. Other genes containing the same domain were later shown to be glycosylphosphatidylinositol(GPI)-anchored variant surface proteins and to be expressed rather in merozoite stages [38].

Merging both clusters and computing a single gene tree confirmed two distinct clades of SAGs for rodent and avian *Eimeria* (Additional file 13). Only one *E. falciformis* gene was found to be conserved across species and clustered with basal *E. tenella* surface antigens which are also conserved across the species infecting chicken (SAGa according to [18]). SAGb and SAGc versions of the domain were not found in *E. falciformis*.

This conserved SAGa version of the domain could very well have a conserved function in the attachment to cells [39] and also be a possible cause of cross-protective immune responses induced by some experimental anti-*Eimeria* vaccines [40]. *T*he function of the SAGa proteins diverged between mammalian and avian *Eimeria* species awaits further investigation.

The largest species-specific cluster of paralogs in *E. falciformis* comprised 14 paralogs without any annotation. A single *Eimeria*-only cluster with 103 hypothetical proteins from *E. tenella* had notably no member from *E. falciformis.* While we cannot infer detailed function of these expanded paralog families, similar gene clusters from other species suggest role in host-parasite interaction.

The restriction of surface antigens to a particular species reflects the high specificity of host-parasite interaction within the genus *Eimeria* and in the Apicomplexa in general. These surface antigens are prime candidates for determinants of the strict preference of the parasites for particular sites within the intestinal tract.

Micronemes, organelles of the apical complex, secrete host interacting molecules early during the invasion of a new host cell. Some of the characteristics (i.e. domain content) of these proteins both in *T. gondii* and *E. tenella* are known (reviewed in [41]). Functional interpretation of our phylogenetic profiling revealed genes likely involved in invasion and egress:

Ortholog clusters restricted to the Coccidia were found heavily enriched for “EF-hand-like” “Epidermal growth factor-like” and “Peptidase S8, subtilisin-related” domains. The latter are found in micronemal proteins of *T. gondii* cleaving other proteins as part of the secretory pathway and during invasion [42]. The GO terms “cyclic-nucleotide phosphodiesterase activity” (GO:0004112) and “calcium ion binding” (GO:0005509) also point towards an involvement in invasion and motility of these proteins. Additionally, ortholog cluster with *E. falciformis* genes annotated as “pyrophosphatase” emerged in the Coccidia. Pyrophosphatase localizes to acidocalcisomes, special apical organelles storing calcium in *T. gondii*[43]. Cyclic nucleotide signaling and calcium-dependent signal transduction are considered a major control element altering cytoskeleton organisation and motility of extracellular stages in order to induce host cell invasion and egress [44].

At the level of the genus *Eimeria*, DNA and protein binding were among the most enriched functions in novel genes. The AP2/ERF domains associated with predicted nucleic acid binding activity for example are known from transcription factors in *T. gondii*[45]*and Plasmodium*[46]*.* According to our analysis we have a lack of orthologous AP2 containing genes but rather the emergence of the majority of these transcription factors at the genus level in *Eimeria*.

Other prominent functions divergent at the genus level were kinase and transferase activity and metal ion binding. Domains like Ankyrin, Armadillo and zinc fingers suggest further diverged control of gene-expression, signaling and protein modification.

We can thus conclude that the calcium signaling components of the invasion mechanism and the majority of the known downstream molecules are conserved throughout the Coccidia. Other aspects of signalling and control mediated by protein-nucleic acid and protein-protein binding, however, are diverged between Sarcocystidae and Eimeriidae and show conservation only within the genus *Eimeria*.

#### Rhophtry kinases

In *T. gondii*, an important role in reprogramming of host cells is ascribed to a largely expanded family of >40 kinases and pseudokinases (RopKs). Many RopKs in *T. gondii* are transported beyond the parasite plasma membrane in order to interact with the host’s immune system [47]. Analyzing kinomes of *T. gondii* and *E. tenella*, Talevich et al. [48] found 41 and 24 RopKs, respectively*.* They later [49] refined a set of 44 Hidden Markov Models to search and classify RopKs across the Coccidia.

**Figure 4:**
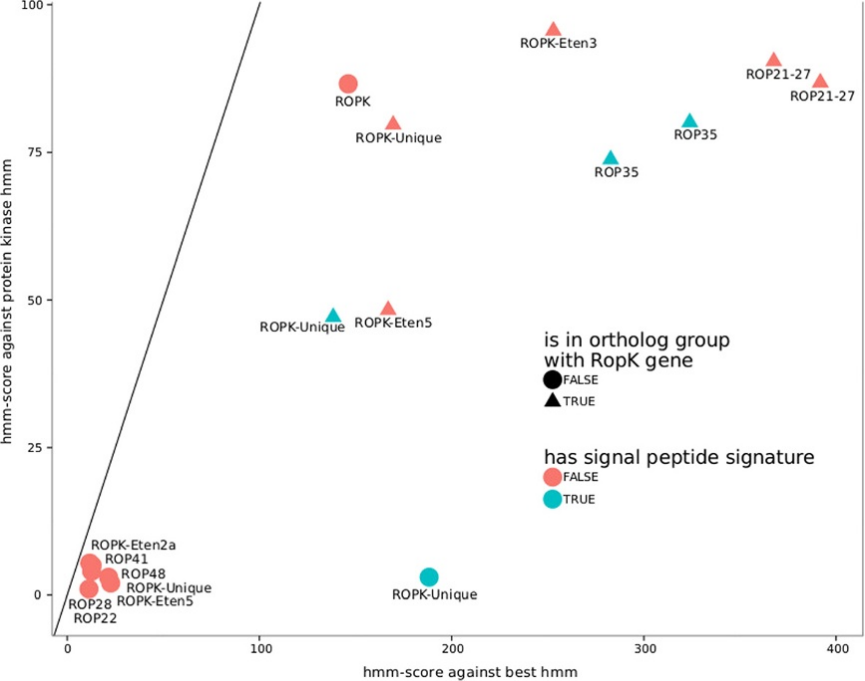
**Identification and grouping of rhoptry kinases.** Rhoptry kinases (RopKs) were searched in the proteome of *E. falciformis* using HMMer3 and hidden markov models (HMMs) for specific RopK families developed by Talevich and Kannan [49]. The figure shows the bitscore of the search against the general protein kinase HMM plotted against the score against best HMM in the database. Candidate rhoptry kinase proteins fall below the diagonal line where general kinases would be found and are labeled with the name of the respective best database HMM. The shape of symbols indicates the presence of a known *T. gondii* rhoptry kinase in the ortholog cluster of the protein, the color highlights the presence or absence of a signal peptide for secretion.

We found orthologs categorized as RopK for 8 genes in the genome of *E. falciformis* (Table 3). A search using the mentioned HMMs yielded 2 additional candidates and grouped all 10 (8 + 2) RopKs in HMM-defined families (Figure 4 and Table 3). Interestingly, the predicted RopKs were substantially enriched in potentially secreted molecules, as four contain a signal peptide for possible secretion. Only three of the other 85 general protein kinases (PF00069; score > 100) contained a secretion signal.

**Table 3:** **Eimeria falcifomis Rhoptry kinases (RopKs)**

gene	Best HMM	Best score	General PK score	***E. tenella*** ortholog	***T. gondii*** ortholog
EfaB_MINUS_17096.g1521	ROPK-Eten3	252.90	95.60	ETH_00005415, ETH_00028835	
EfaB_MINUS_32658.g2475	ROPK-Eten5	167.00	48.30	ETH_00002510	
EfaB_MINUS_42996.g2710	ROP35	323.90	80.10	ETH_00005905	TGME49_104740
EfaB_MINUS_720.g57	ROPK-Unique	169.60	79.70	ETH_00005190	ROP18^++^
EfaB_PLUS_15899.g1411	ROPK	146.20	86.60		
EfaB_PLUS_24117.g1969	ROPK-Unique	188.30	3.00	ETH_00013325*	
EfaB_PLUS_33184.g2393	ROPK-Unique	138.40	47.10	ETH_00005170	
EfaB_PLUS_47595.g2679	ROP21-27	367.60	90.40		TGME49_113330
EfaB_PLUS_7742.g778	ROP21-27	391.80	86.80	ETH_00014495	TGME49_063220
EfaB_PLUS_8664.g829	ROP35	282.70	73.80	ETH_00026495	

For putative *E. falcifomis* RopKs the family categorization obtained searching the HMMs developed by Talevich et al. [49] is given (best HMM). The score against this HMM can be compared with the score against the general protein-kinase-like profile (PKL score) to distinguish RopKs from universal kinases (see also Figure 4). Finally orthologs from *E. tenella* and *T. gondii* are given. The *E. tenella* ortholog marked with * is not included in the RopKs described by Talevich et al. [48], therefore EfaB_PLUS_24117.g1969 is not counted as having a RopK ortholog. Rop18 (marked with ^++^) is not found in ortholog clusters. The *E. falciformis* gene, however, shows sequence similarity to Rop18-III from *T. gondii* (GI: 117574634).

Close paralogs of RopK-family genes are often found in tandem repeated clusters within the genome, as shown for *T. gondii* Rop5 (Behnke et al., 2011). We can however rule out that the low number of *E. falciformis* RopKs is an artifact of sequence assembly merging highly similar paralogs into single gene models: *E. falciformis* RopKs are neither significantly closer to contig borders than other genes nor did we observe elevated sequencing coverage for the corresponding genomic regions. The extremely low number of RopKs in *E. falciformis* thus might indicate a lower number of these proteins present in rodent than in avian *Eimeria* parasites.

Rop18 is a highly polymorphic pathogenicity factor, whose allelic types determine the virulence of parasite strains [20]. Rop18 of the virulent Type I *T. gondii* strain associates with the parasitophorous vacuole membrane of tachyzoites, where it inactivates host defence proteins, the Immunity Related GTPases (IRGs) by phosphorylation [50]. We did not find orthologs of Rop18 in the genome of *E. falciformis* and searches with the respective HMM did not yield significant results. One of our predicted RopKs, however, showed some similarity to Rop18 that did not lead to inclusion in an ortholog cluster. The Rop18 similar gene was not in an ortholog cluster with an *E. tenella* gene and also the similarity to *T. gondii* Rop18 was higher than to any *E. tenella* gene. Recent studies show that Irgb6 is not localized to the parasitophorous vacuole of intracellular *E. falciformis* sporozoites [11]. It remains to be tested whether *E. falciformis* needs a Rop18-like protein to release its parasitophorous vacuole from the defence mechanism executed by the IRG-system. Future studies will address if other functionally related proteins inactivate murine components in infections with *E. falciformis* by phosphorylation.

Neither orthologs, HMM hits or basic similar sequences were found for Rop16, another pathogenicity factor secreted by *T. gondii* to down regulate immune responses [51]. It is tempting to speculate that the comparatively low number of RopKs in Eimeria is due to the fact that these parasites are specialized to a narrow spectrum of gut epithelial cells of a given host species, requiring a less diverse set of molecules to interact with the host immune system.

Additionally, *Eimeria* might not need to manipulate some aspects of immune responses, as these parasites typically exploit their hosts for a limited time frame, whereas *T. gondii* establishes life-long infections. In contrast, the large set of RopKs in *T. gondii* would be needed to allow infection and modulation of a large variety of host cells over a long period, as this parasite infects virtually every nucleated cell and intensively modulates host immune responses.

### Metabolic potential

Intracellular parasites like *E. falciformis* are expected to possess a rewired metabolism with multiple heterotrophies, as they thrive on nutrients of host origin. To identify such metabolic peculiarities, we assessed the metabolic potential of *E. falciformis* with a combination of methods employing simple comparisons of domain and metabolic enzyme annotations but also automated genome scale metabolic reconstruction based on MetaCyc [52] and KEGG [53] pathways.

387 distinct EC numbers (in full detail to the 4th digit) for metabolic enzyme activities were obtained for 670 genes: for 301 through domain associations in InterProScan, 220 by ortholog assignment to *T. gondii* LAMP annotations [54] and 193 by SwissProt sequence similarity searches while controlling for domain agreement (Additional file 14).

We created two different versions of EfaCyc, our MetaCyc- derived model of the *E. falciformis* metabolism: One more accurate but incomplete and one less accurate but more comprehensive model. The comprehensive EfaCyc metabolic reconstruction comprised 271 pathways and 688 enzymes. The smaller reconstruction pruned for pathways unlikely to be present based on taxonomy listed only 125 pathways with 671 enzymes. KEGG analysis predicted 90 pathways with 325 different enzymes encoded by 365 genes.

Transporters constitute an interface between host and parasite metabolic networks. We therefore investigated especially transport annotations and also predicted transport reactions in our metabolic reconstruction [55]. Our comprehensive EfaCyc reconstruction predicted 44 transporters for 29 transport reactions; the taxonomically pruned reconstruction recovered only 5 transport reactions and 24 transporters. KEGG analysis found 6 different transmembrane transport reactions and 8 distinct transporters.

**Figure 5:**
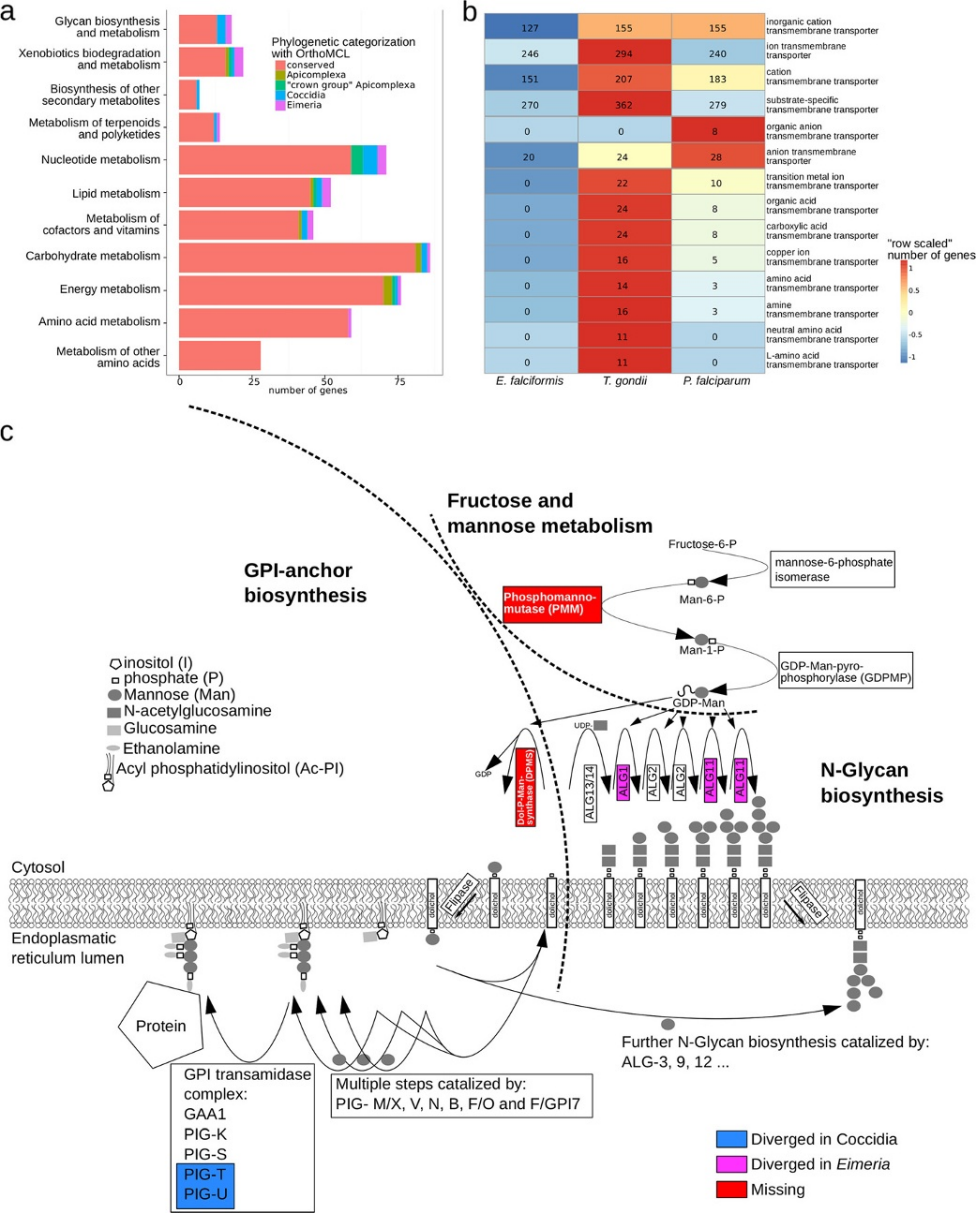
**Phylogenetic profile of metabolic enzymes and transporters. a)** Phylogenetic profiles of *E. falciformis* genes were determined based on OrthoMCL clustering results and are shown separately for KEGG metabolic modules (groups of pathways). These KEGG annotations were derived with the KEGG Automatic Annotation Server KAAS and most metabolic enzymes are highly conserved. Bars for different modules are ordered by the proportion of novel genes at the genus *Eimeria* and subclass Coccidia level (see Figure 3). **b)** Genes annotated with Gene Ontology (GO) terms representing transporters (GO:0005215 “transporter activity” and descendants) and underrepresented in *E. falciformis* are compared for their representation in *T. gondii* and *P. falciparum.* Numbers of genes for a specific term were normalized with the total number annotations in a species and compared. Cells depict these normalized numbers of genes. Colors are additionally scaled by the total number of genes annotated for the term (row). **c)** Intersection of the Fructose and Mannose Metabolism, GPI-anchor and N-Glycan biosynthesis pathways. Colors are given for missing enzymes or enzymes with restricted phylogenetic profile.

Genes with an annotated function as transporters (GO:0005215 “transporter activity” and descendants) are more prevalent in *T. gondii* than in *E. falciformis* and slightly more prevalent in *P. falciparum* than in *E. falciformis* (Figure 5b). Especially enriched in *T. gondii* relative to *E. falciformis* were the terms amino acid-, organic acid-, organic anion-, carboxylic acid-, amine- and copper ion- transmembrane transporter activity (GO:0015171, GO:0005342, GO:0008514, GO:0046943, GO:0005275 and GO:0005375). The corresponding *T. gondii* genes had twelve transport-associated domains not present in any *E. falciformis* gene: amino acid, Ctr-copper, ABC family E, UDP-galactose, and magnesium transmembrane-, but also intraflagellar transporter domains. These suggest a richer repertoire of transmembrane transporters in *T. gondii* than in *E. falciformis.* The reduced host-range of the Eimeriidae seems a plausible reason for the loss of transporters, which would be linking host and parasite metabolic systems.

“Drug/metabolite transporter” (IPR000620), “Organic anion transporter polypeptide OATP” (IPR004156) and “Cytochrome b-561/ferric reductase transmembrane” (IPR006593) were the only transport associated domains found in *E. falciformis* that are absent from *T. gondii,* as an analysis species specific domains demonstrated (Figure 2). The drug/metabolite transporter domain absent from *T. gondii* is found in other Apicomplexa*,* the anion transporter polypeptide OATP domain is - apart from *E. falciformis* - only found in Trypanosomes and otherwise restricted to the Metazoa, the ferrireductase is only found outside the Apicomplexa and in *E. falciformis.* Based on the latter domain EfaCyc predicted a transmembrane ascorbate ferrireducatase. The proteins (2 isoforms) contain 4 and 6 transmembrane helices and are predicted to transport electrons through single membrane acting as diheme cytochrome. This is the only transporter prediction in EfaCyc which does not have an ortholog in *T. gondii*.

Iron uptake *via* lactoferrin is especially relevant in mucosal tissue, where lactoferrin is the canonical iron chelator. The transmembrane ferrireductase could be part of an alternative route for iron uptake, as part of which iron is reduced to the soluble ferrous form. A similar protein is required in *Leishmania amazonensis* for iron uptake from lactoferrin and replication within macrophages [56]. We found a *E. falciformis* protein similar to the *Leishmania* iron permease *Lit1* as a candidate for the ferrous iron transporter required to take up the produced ferrous iron.

We were able to identify three diversified Coccidia-specific ABC-transporters, as the KEGG module “Membrane transport” was overrepresented in genes both novel and expanded in the Coccidia. While eukaryotic ABC transporters translocate a variety of endogenous metabolites across extra- and intracellular membranes, they are also prominent for their role in xenobiotic detoxification and drug resistance [57].

Pathways in the KEGG module “Glycan biosynthesis and metabolism” were enriched in both ortholog clusters novel in the genus Eimeria and in the Coccidia (Figure 5a, Additional file 8). We inferred that different aspects of glycan biosynthesis show different conservation patterns.

N-Glycan biosynthesis represented by the genes beta-1,4-mannosyltransferase (ALG1; EC:2.4.1.142) and alpha-1,2-mannosyltransferase (ALG11; EC:2.4.1.131) is found in diverged ortholog groups specific to the genus Eimeria. In addition, “FAD-linked oxidase, C-terminal” (IPR004113) domains were found in three *E. falciformis* genes but were absent from *T. gondii* and all other Apicomplexa outside the genus *Eimeria*. The phylogenetic profile of the latter two domains and the corresponding gene-families suggests a lateral gene transfer. KEGG analysis predicted the genes containing FAD-linked oxidase domains to be part of peptidoglycan synthesis acting as UDP-N-acetylmuramate dehydrogenases (EC 1.3.1.98).

On the other hand enzymes of the pathways “Glycosylphosphatidylinositol(GPI)-anchor biosynthesis” and “Mucin type O-glycan biosynthesis” show less restricted conservation. Two subunits of the GPI transamidase complex, Phosphatidylinositol glycan, class T (PIGT) and phosphatidylinositol glycan, class U (PIGU), and N-acetylgalactosaminyltransferase (GALNT; EC:2.4.1.41) are diversified in the Coccidia but conserved throughout both Eimeriidae and Sarcocystidae. Given the between species divergence of the surface antigens it is reassuring that at least enzyme subunits involved in the synthesis of their membrane anchors are conserved within the Coccidia.

However there were also some differences in this respect between *Eimeria* and other Coccidia. We profiled ortholog clusters with otherwise complete apicomplexan distribution absent from all *Eimeria* species and scrutinized this list with basic similarity searches. Phosphomannomutase (PMM; EC:5.4.2.8) and dolichol-phosphate-mannose synthase (DPMS; EC:2.4.1.83) are prominent among the genes lost, due to their proximity in the pathways of mannose (Man) metabolism and N-glycan synthesis. Both enzymes are needed to produce activated Dol-P-Man, which serves as a substrate for N-glycan biosynthesis and the formation of glycoconjugates, among them GPI-anchored proteins.

Our results suggest a rewiring of the glycosylation pathways in the Eimeriidae as different enzymes are present, absent or show sequence divergence compared to *T. gondii* or other Apicomplexa*.* It will be interesting whether these differences are more pronounced in the synthesis of N-glycans than of GPI-anchors, as suggested by our analysis.

We have used the presented reconstructions of the *E. falciformis* metabolism with the clear scope to identify missing or additional metabolic modules and focused especially on transporters. A refinement of the constructed metabolic network and a combination with gene expression data from host and parasite, will allow further analysis of heterotrophies related to the uptake of host metabolites. First candidate pathways with missing intermediate reactions, divergent enzymes and enzymes acquired by lateral transfer include malate metabolism, glycosylation pathways and iron uptake.

## Conclusions

We presented the complete genome sequence of *Eimeria falciformis,* a single-host parasite of the mouse. We reported on the largely contiguous reconstruction of the first Eimeria genome from a host other than chicken and predicted a reduced and remodeled set of protein coding genes in comparison to the model parasite *Toxoplasma gondii* (5879 vs. 8115).

Specifically, *E. falciformis* possesses fewer rhoptry kinases (important virulence factors) and a reduced number and diversity of transmembrane transporters in comparison to *T. gondii.* The difference in genome size and protein-coding gene set complexity could be a cause or consequence of the differences in host range (specialist vs. generalist) and tissue tropism (narrow vs. broad)*.*

We observed that gene families that are involved in intracellular signaling, control of gene expression and protein modification are conserved at the level of the genus *Eimeria.* However, the same families diverged within the Coccidia across the two large families, the Eimeriidae and the Sarcocystidae. Mediators of invasion and motility, as well as the calcium signalling component controlling them, are however conserved and expanded in the Coccidia.

We are certain that *E. falciformis* with its accessible and traceable laboratory host has great potential to become a unique model for comparative host-parasite interaction analysis in Coccidia and beyond. Especially, research on avian *Eimeria* species with their economic importance for the poultry industry will greatly benefit from this newly established satellite model system.

We have contributed the molecular blueprint for future research on the life cycle in *Eimeri*a and dynamics of infection in the laboratory mouse.

## Methods

### Sample preparation for genomic sequencing

Female NMRI mice were infected with 200 sporulated oocysts as described in Schmid et al. [10]. At days 8 to 10 post infection faeces were collected, washed and treated with 2.5% potassium bichromate. Oocysts were allowed to sporulate for one week. For purification of sporulated oocysts, faeces were sterilized and recovered with sodium hydrochloride as described by Hoffmann and Raether (1990) and Hosek et al. (1988). Sporocysts were isolated according to the method of Kowalik and Zahner (1999) with slight modifications. Briefly, not more than 5 million sporulated oocsyts were resuspended in 0.4% pepsin-solution (Applichem), pH 3 and incubated at 37°C for 1 hour. Subsequently, sporocysts were isolated by mechanical shearing using glass beads (diameter 0.5 mm), recovered from glass beads by intense washing and seperated from oocyst cell wall components by centrifugation at 1800 g for 10 min. Sporocysts were suspended in lysis buffer and were shock frozen 3 times in liquid nitrogen. Proteins were digested with proteinase K for 45 min at 65°C. Nucleic acids were recovered by phenol/chloroform extraction and RNA was digesetd using RNase A. Genomic DNA was recovered by phenol/chloroform and chloroform extraction, followed by ethanol precipitation. The DNA pellet was resuspended in an appropriate volume of VE-water. DNA quality was assessed using an Agilent Bioanalyzer (Agilent, Santa Clara, United States). 5 μg of high quality DNA were used to prepare a paired-end DNA library according to the manufacturer’s protocol (Illumina). Additionally, 50 μg high quality DNA were subjected to a large insert mate pair preparation protocol (Illumina).

### Sample preparation for RNA-Sequencing

Sporozoites were isolated from sporocysts by excystment. For this, sporocysts were incubated at 37°C in DMEM containing 0.04% tauroglycocholate (MP Biomedicals) and 0.25% trypsin (Applichem) for 30 min. Sporozoites were purified by the method of Schmatz et al. [58]. Unsporulated oocysts were purified as described above, except for collecting the faeces every 12 hours to prevent oocysts from sporulating. For each library of merozoite/gametocyte stages the cecum of at least 3 NMRI mice was isolated. Epithelial cells were isolated as described in Schmid et al. [10].

Total RNA was isolated from infected epithelial cells, sporozoites and unsporulated oocysts using Trizol according to the manufacturer’s protocol (Invitrogen). High quality RNA was used to produce an mRNA library using the Illumina’s TruSeq RNA Sample Preparation guide.

### Assembly and estimation of genome completeness

For genomic DNA a conventional paired-end sequencing library (fragment size 370 nt, read length 2 × 100 nt) and a large-insert mate pair library (insert size 2 kb, read length 2 × 50 nt) were sequenced. Sequencing adapters were clipped from all short reads using Flexbar [59]. Both read sets were simultaneously assembled with Velvet 1.2.03 [60] on a large memory machine. Apicoplast and mitochondrial genomes were reassembled from reads of first-pass contigs that aligned to organellar genomes of other Eimeria species.

We evaluated the completeness of the genome assembly by testing for the presence of a highly conserved eukaryotic core gene set (n = 248) using CEGMA [61] (Additional file 15). Additionally we evaluated the number of pan-apicomlexan genes based on orthologous assignment (see below) missing from our assembly. As a benchmark we performed the same analyses with publicly available datasets from EupathDB [13, 62] (version 7.3; Additional file 16) and an *Eimeria maxima* draft genome available in EmaxDB [17].

### Annotation of genomic regions

We annotated transfer RNAs (tRNAs) with tRNA-Scan [63] and ribosomal RNAs (rRNAs) using BLAST against the Silva database [64]. Simple Sequence Repeat (SSR) regions were identified using the MIcroSAtellite identification tool MISA (http://pgrc.ipk-gatersleben.de/misa/) and non-SSR Regions using the RepeatScout [65] and RepeatMasker [66] pipeline. Additionally, we identified transposable elements using TransposonPSI (http://transposonpsi.sourceforge.net) against profiles for proteins from (retro-) transposon families.

For annotation of protein coding genes Illumina TruSeq mRNA libraries of multiple life cycle stages were sequenced in the 2 × 100 nt format. All short reads were quality-controlled and splice-aligned to the genome sequence using TopHat 1.4.0 [67]. Transcript structures were inferred with the Cufflinks software [68] and used to train the gene-finding software Augustus [69]. Based on this training-set and additionally incorporating expression evidence from RNA-Seq, we used Augustus to predict gene-structures, coding regions and untranslated regions (UTRs).

### Functional annotation

InterProScan (version 5 RC4) [26] was used to predict protein domains for *E. falciformis* and to allow an unbiased comparison regarding annotation software also for *T. gondii*. InterPro domain accessions were compared for the two proteomes and enrichment in one species over the other was analyzed using Fisher’s exact test.

Annotations with Gene Ontology (GO) [27] terms and Enzyme Comission (EC) numbers were obtained from domain architecture if possible. Additionally, EC numbers for *T. gondii* genes were obtained from the hand-curated Library of Apicomplexan Metabolic Pathways (LAMP) database [54]. Based on orthologous annotations (see OrthoMCL clustering below) these enzyme annotations were transferred to the *E. falciformis* proteome.

As third source of enzyme annotations we transferred functions from a BLAST search vs. SwissProt (e-value threshold 1e-5). We limited this similarity-based annotation to cases for which at least three quarters of the InterPro accessions for the Swissprot hit and the InterProScan of our *E. falciformis* proteins agreed.

### Orthologous clusters and comparative analyses

OrthoMCL [32] was used with BLAST e-value cut-off of 1e-5 and inflation value of 1.5 to cluster *E. falciformis* proteins with the proteomes of other Apicomplexa with fully sequenced genomes (the same species used for genome comparisons (Additional file 16). OrthoMCL tries to separate out-paralogs from in-paralogs: paralogous pairs that emerged after a speciation event are contained in the same cluster whereas paralogous pairs that were present prior to a speciation event will be separated into two clusters. This usually produces a rather fine-grained clustering.

Based on these clusters proteins were phylogentically stratified and gene-set enrichment was performed for GO-terms using the R-package TopGO and InterPro domains using Fisher’s exact test.

Additionally, we constructed protein alignments for all OrthoMCL clusters using Muscle (version 3.8.31) [70]. Alignments were trimmed using trimAl (version 1.2rev59 with trimming parameters set to “automatic”) [71] and phylogenetic trees were reconstructed using PhyML (version 20120412) [72] using the WAG model for amino-acid replacement and BIONJ as starting tree.

We excluded all *C. parvum* and *E. maxima* genes from our tree-reconciliation approach because for the first the species phylogeny in relation to the other species is not fully cleared and for the latter many alignments seemed truncated and incomplete. The remaining gene trees were reconciliated with the species tree using ranger-DTL (version ranger-dtl-U.linux 1.0) [73] with transfer cost set to 10, duplication cost to 2 and the cost of loss to 1. Clusters were classified as expanded if duplication events were found at a certain node. TA4 surface antigen domain containing protein alignments were built using hmmalign of HMMER3 [74].

For SAGs, fructose-bisphosphate aldolase and membrane alanyl aminopeptidase peptidases phylogenetic trees were built using MrBayes v3.2.1 [75] with the mixed models setting for amino acid replacement set in priors. MCMC samples were obtained every 100 generations from 4 chains starting with random trees and running for 1,000,000 generations generations. Burnin was set to 2,500 samples, discarding the first 25% of trees. Bayesian and maximum likelihood phylogenetic trees were compared using SumTree [76].

### Pathway analyses

EC numbers and protein coordinates were used as input to the PathwayTools Pathologic suite V17.0 [77] and pathways were predicted based on the MetaCyc database [52]. The transporter prediction module of PathwayTools [58] was used to predict transmembrane transporters and the presence of orthologs of these transporters was checked overlaying these predicitions with OrthoMCL clustering data.

Additionally we used the KEGG automatic annotation server (KAAS) [78] to obtain Kegg onthology (KO) identifiers and associated Kyoto Encyclopedia of Genes and Genomes (KEGG) [56] pathways. We tested overrepresentation of metabolic pathways in different gene-sets using fisher’s exact tests.

## Ethical approval

Production of *E. falciformis* and collection of infected mouse tissues are approved by the Landesamt für Gesundheit und Soziales Berlin (H0098/04).

## Authors’ information

Emanuel Heitlinger and Simone Spork as joint first authors

Richard Lucius and Christoph Dieterich as joint last authors.

## Electronic supplementary material

Additional file 1: **Genome coverage estimated from mapping (figure).** Coverage from two different sequencing libraries for individual contigs of the final *E. falciformis* genome assembly - The read depth (coverage) estimated by mapping of raw sequencing reads back to the final assembly is given for the 2 × 100 bp short insert library on the y-axis and the 2 × 50 bp long insert (mate pair) library on the x axis. Mitochondrial and apicoplast reconstructions are highlighted in green and red color respectively, their coverage is clearly elevated. (PDF 56 KB)

Additional file 2: **Reassembly of the apicoplast genome as compared to** ***E. tenella*** **(figure).** Apicoplast derived reads were reassembled using velvet and the resulting contigs were compared to the *E. tenella* complete apicoplast genome [23]. Sequence similarity and annotated features are visualized in the *E. tenella* apicoplast genome. The inferred synteny was used to reconstruct the *E. falciformis* apicoplast genome partitioning or reversing sequence and adding undetermined bases where necessary. (PDF 292 KB)

Additional file 3: **GC content of different genome features for E. falciformis (figure).** For genome features annotated in the context of protein-coding genes using Augustus [69], the distribution of the per-feature GC content is given. While exons and coding sequence exons show a slightly elevated GC, introns have a lower GC. (PDF 25 KB)

Additional file 4: **Structural comparison of protein coding genome features of Apicomplexa (figure).** Protein coding features of apicomplexan genomes obtained from EupathDB (see Additional file 16 for exact versions) were compared with gene-predictions for *E. falciformis* obtained combining expression evidence (RNAseq) with *ab initio* gene-finding using Augustus [72]. Panels show the distribution of a) the overall length of genes, b) the size of individual exons and c) introns and d) the number of coding sequence exons per gene. (PDF 144 KB)

Additional file 5: **Interpro annotation of** ***E. falciformis*** **genes (csv table).** *E. falciformis* genes were annotated with InterproScan (version 4 RC5) [26]. The table is simplified and lists only distinct Interpro identifiers (IPR) for all annotated genes. (CSV 658 KB)

Additional file 6: **GO annotations of** ***E. falciformis*** **genes (csv table).** GO terms were obtained for *E. falciformis* genes from domain based annotations. The table lists all GO terms obtained for the annotated genes. (CSV 385 KB)

Additional file 7: **Interpro annotation of** ***T. gondii*** **(csv table).** *T. gondii g*enes were annotated with InterProScan (version 4 RC5) to obtain annotations directly comparable to *E. falciformis.* The table is simplified and lists only distinct InterPro identifiers (IPR) for all annotated genes. (CSV 312 KB)

Additional file 8: **Enrichment analyses for ortholog clusters novel in certain clades of the apicomplexan phylogeny (figure containing tables).** Gene sets identified as novel in certain clades of the apicomplexan phylogeny (Figure 3), were searched for enriched function using F-tests. The tables list enrichment results for GO molecular function, biological process, InterProScan domains and KEGG pathways. Table columns indicate IDs and names of the annotation, how often the annotation was found in the all ortholog clusterd *E. falciformis* genes, how many genes would be expected by chance based on this and the size of a gene-set and the p-value for the enrichment. (PDF 48 KB)

Additional file 9: **Enrichment analyses for ortholog clusters expanded in certain clades of the apicomplexan phylogeny (figure containing tables).** Genes sets contained in ortholog clusters expanded at certain clades of the apicomplexan phylogeny (Figure 3), were searched for enriched function using F-tests. The tables list enrichment results for GO molecular function, biological process, InterProScan domains and KEGG pathways. Table columns indicate IDs and names of the annotation, how often the annotation was found in the all gene/species-tree reconciliated *E. falciformis* genes, how many genes would be expected by chance based on this and the size of a gene-set and the p-value for the enrichment. (PDF 66 KB)

Additional file 10: **Pairwise phylogenetic distance genes expanded at different nodes of the apicomplexan phylogeny (figure).** Boxplots (overlayed with single datapoints) are given for the the maximal phylogenetic distance of two genes in an expanded ortholog clusters as estimated by the WAG model in PhyML [72]. Ortholog clusters are grouped by expansions at different nodes of the apicomplexan phylogeny. (PDF 38 KB)

Additional file 11: **Phylogenetic tree for Fructose bisphosphate aldolase (figure).** The ortholog cluster containing *E. falciformis* genes annotated as fructose bisphosphate aldolase (FBA) was found to be independently expanded in both Eimeriidae and Sarcocystidae. Sequences for the ortholog group were aligned using muscle, trimmed with trimAl and a phylogenetic tree was inferred with Mister Bayes and PhyML. Labels below branches give the number of bootstrap replicates supporting the clade (out of 100), labels above branches indicate its bayesian posterior probability. For branches with 100% bootstrap support or a posterior probability of 1, labels are omitted. After gene duplication in the Sarcocystidae (FBA Sarco), both paralogous copies were retained in *T. gondi*i and *N. caninum.* The same fate was experienced by the independently duplicated genes in *Eimeria* (FBA *Eimeri*a), *E. tenell*a, *E. falciformis* and *E. maxim*a each retained their paralogs. (PDF 32 KB)

Additional file 12: **Phylogenetic tree for M1-amylopeptidase (figure).** The ortholog cluster containing *E. falciformis* genes annotated as M1-amylopeptidase (M1 AP) was found to be independently expanded in both Eimeriidae and Sarcocystidae. Sequences for the ortholog group were aligned using muscle, trimmed with trimAl and a phylogenetic tree was inferred with Mister Bayes and PhyML. Labels below branches give the number of bootstrap replicates supporting the clade (out of 100), labels above branches indicate its bayesian posterior probability. For branches with 100% bootstrap support or a posterior probability of 1 labels are omitted. A first duplication common to the Coccidia, preceding the split of the Sarcocystidae and Eimeriidae created two copies of the gene. In one of the resulting subclades (M1AP -A) *N. caninu*m and *T. gondi*i genes were conserved in one copy, the *E. tenell*a gene was lost and the *E. falciformi*s gene duplicated. In the other subclade (M1AP - B) the gene was further duplicated after the split of the Sarcocystidae and Eimeriidae leaving two *T. gondii* paralogs after the loss of one ortholog in *N. caninu*m. Similarly, in the Eimeriidae after initial duplication one of the paralogs was lost in *E. tenell*a, and the *E. falciformi*s paralog expanded in an additional duplication. (PDF 33 KB)

Additional file 13: **Independent expansion of surface antigens.** Surface antigen domain (TA4) containing genes are enriched in both *E. falciformis* and *E. tenella* expanded gene families. Phylogenetic trees were constructed based a HMM guided alignment using MrBayes and PhyML and merged. The nodes separating avian and rodent SAG clades are highlighted with stars, within these supporting bootstrap replicates (from 100) and bayesian posterior probabilities are given. This tree confirms the independent expansion of two clades in *E. falciformis* and *E. tenella* and further highlights one *E. falciformis* gene with higher cross-species similarity to avian than rodent *Eimeria*. (PDF 35 KB)

Additional file 14: **Enzyme Commission (EC) annotations from different sources (csv table).** The table lists annotations with EC numbers for *E. falciformis* genes, the source for these annotations (see Methods for details) and the level of detail (to which digit in the EC hierarchy) of the corresponding annotation. (CSV 40 KB)

Additional file 15: **Evaluation of genome assembly completeness (figure).** Apicomplexan genomes were searched for HMMs of Core Eukaryote Genes (CEGs) using CEGMA [61]. Parra et al. [79] divided these CEGs into four groups according to the degree of conservation observed in the pairwise alignments (4 being the most conserved group, 1 the least). Apicomplexan genome is general show a high divergence of CEGs visible in the low recovery of less conserved groups. CEGs for which proteins are longer than 70% of the corresponding HMM alignment are recognized as fully covered, others as partially covered. Group 4, expected to be fully present even in the reduced genomes of Apicomplexa is with 97% (63 genes) represented nearly completely in our assembly. The representation of less conserved groups 1–3 compares well with the high quality genomes of *Plasmodium falciparum*[80] and *T. gondii*[13]. (PDF 18 KB)

Additional file 16: **Genome sources used (xls table).** The species and the associated genome information used in various comparative genomics analyses. Columns indicate: The whole genome sequence (in nucleotide fasta format) as it was used to estimate genome completeness (CEGMA). The annotation files (in gff format) used to compare basic genomic features and the predicted proteins (in amino acid fasta format) used for ortholog clustering (OrthoMCL) are followed by the name of the database these data was obtained from. (XLS 9 KB)
