# Supplementary figures and images for: The genome of Eimeria falciformis - reduction and specialization in a single host apicomplexan parasite
Source: BMC Genomics. 2014 Aug 20;15(1):696. doi: 10.1186/1471-2164-15-696 (PMC4287421; doi:10.1186/1471-2164-15-696)

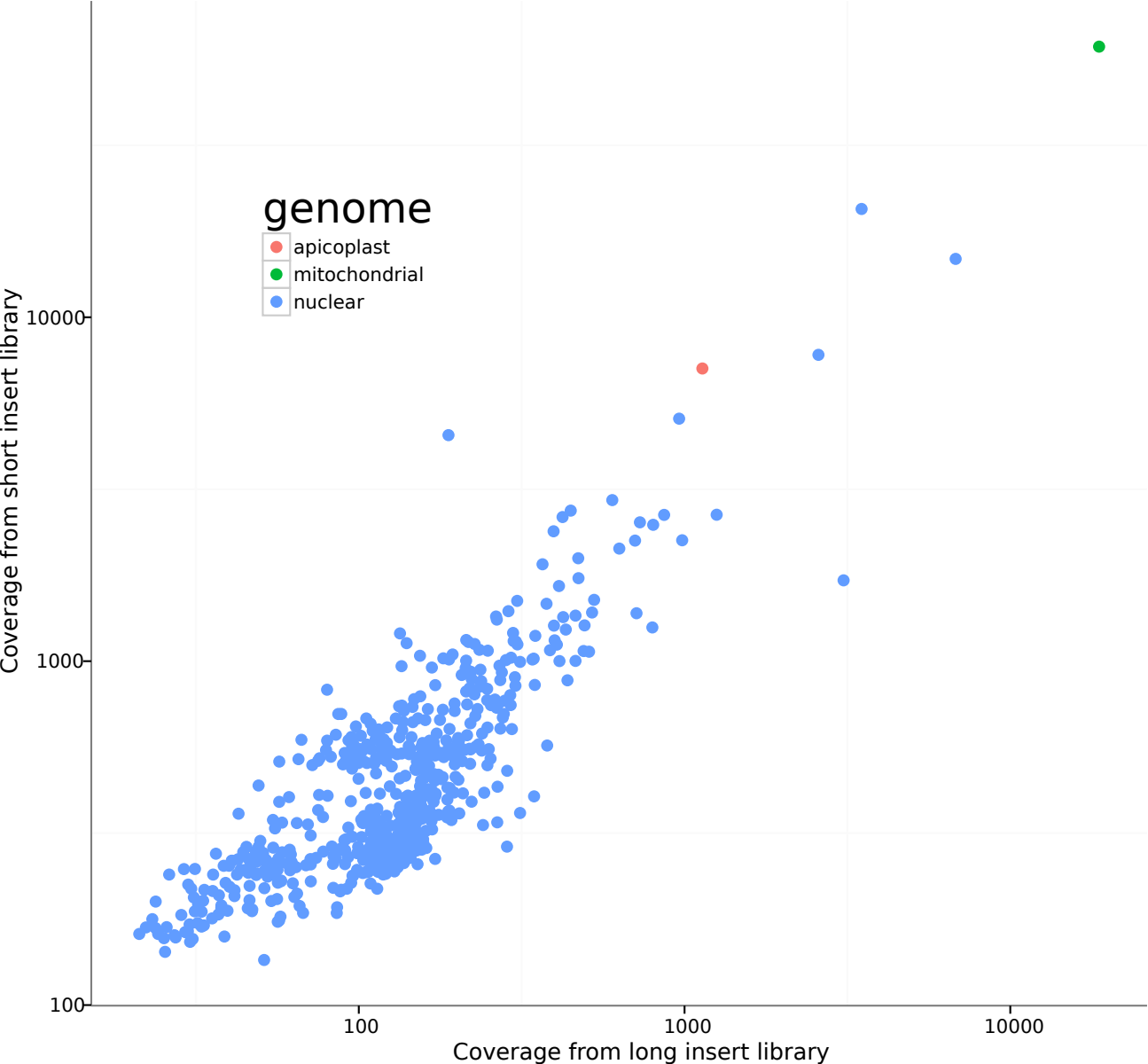

Supplement: Supplementary file 1 — Additional file 1: Genome coverage estimated from mapping (figure). Coverage from two different sequencing libraries for individual contigs of the final E. falciformis genome assembly - The read depth (coverage) estimated by mapping of raw sequencing reads back to the final assembly is given for the 2 × 100 bp short insert library on the y-axis and the 2 × 50 bp long insert (mate pair) library on the x axis. Mitochondrial and apicoplast reconstructions are highlighted in green and red color respectively, their coverage is clearly elevated. (PDF 56 KB) [file 12864_2014_6777_MOESM1_ESM.pdf]

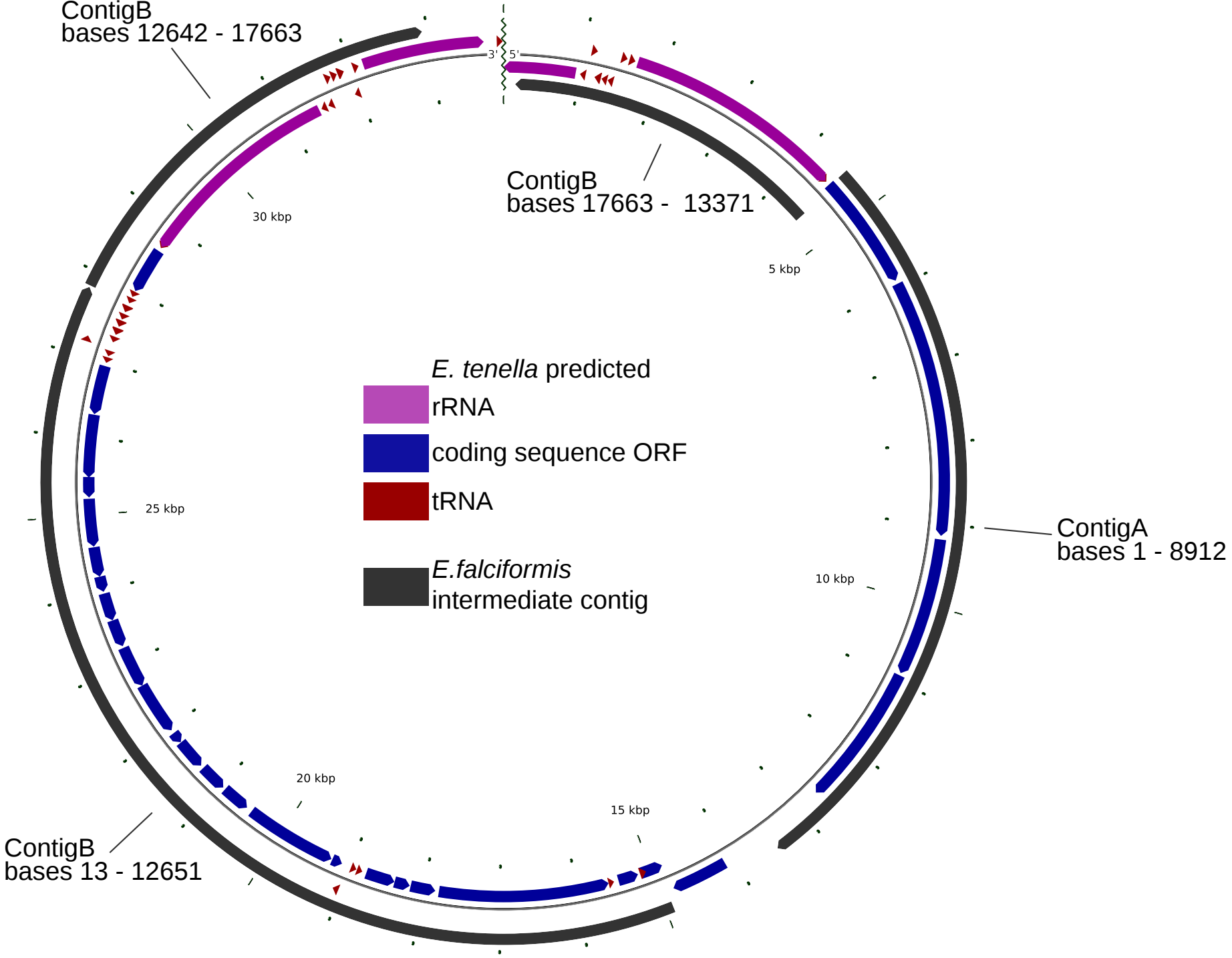

Supplement: Supplementary file 2 — Additional file 2: Reassembly of the apicoplast genome as compared to E. tenella (figure). Apicoplast derived reads were reassembled using velvet and the resulting contigs were compared to the E. tenella complete apicoplast genome [23]. Sequence similarity and annotated features are visualized in the E. tenella apicoplast genome. The inferred synteny was used to reconstruct the E. falciformis apicoplast genome partitioning or reversing sequence and adding undetermined bases where necessary. (PDF 292 KB) [file 12864_2014_6777_MOESM2_ESM.pdf]

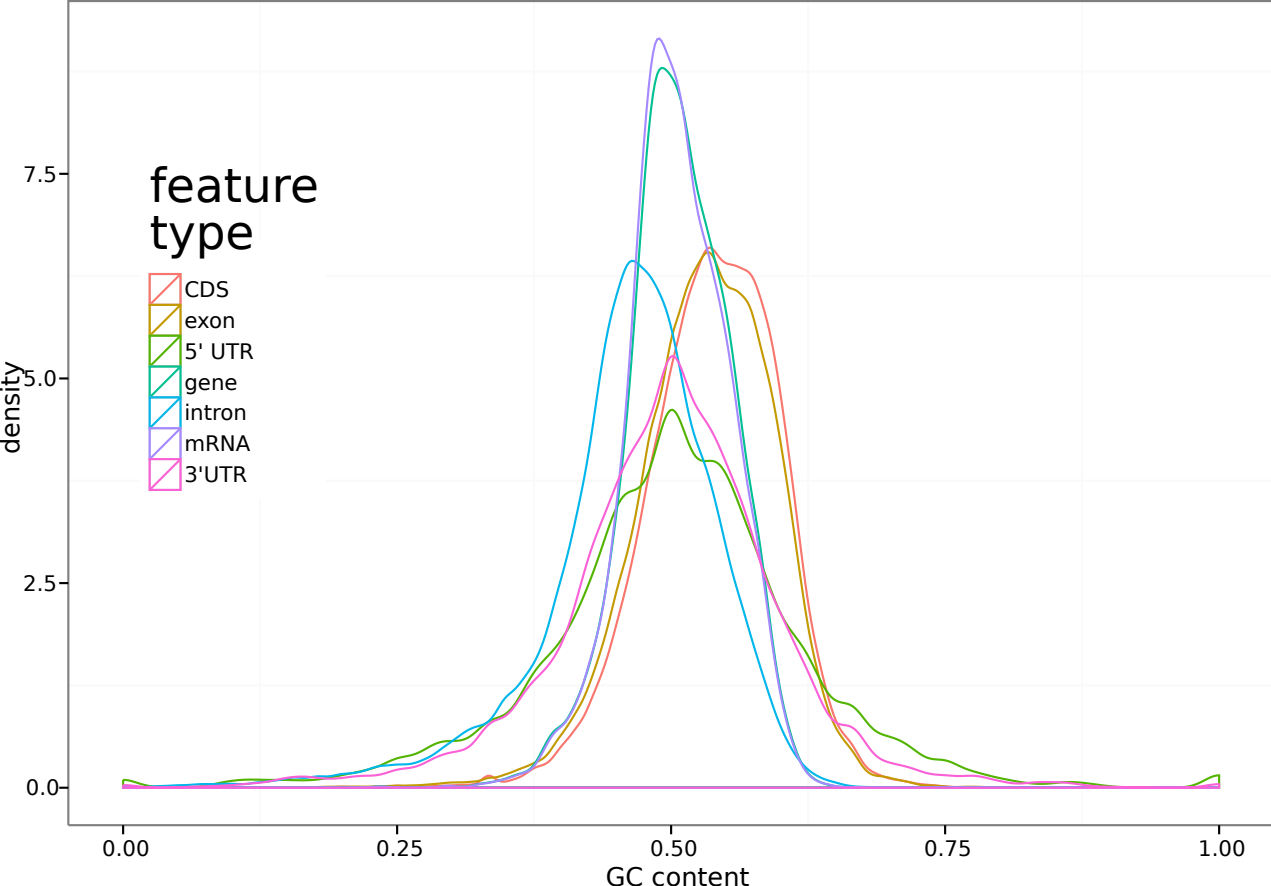

Supplement: Supplementary file 3 — Additional file 3: GC content of different genome features for E. falciformis (figure). For genome features annotated in the context of protein-coding genes using Augustus [69], the distribution of the per-feature GC content is given. While exons and coding sequence exons show a slightly elevated GC, introns have a lower GC. (PDF 25 KB) [file 12864_2014_6777_MOESM3_ESM.pdf]

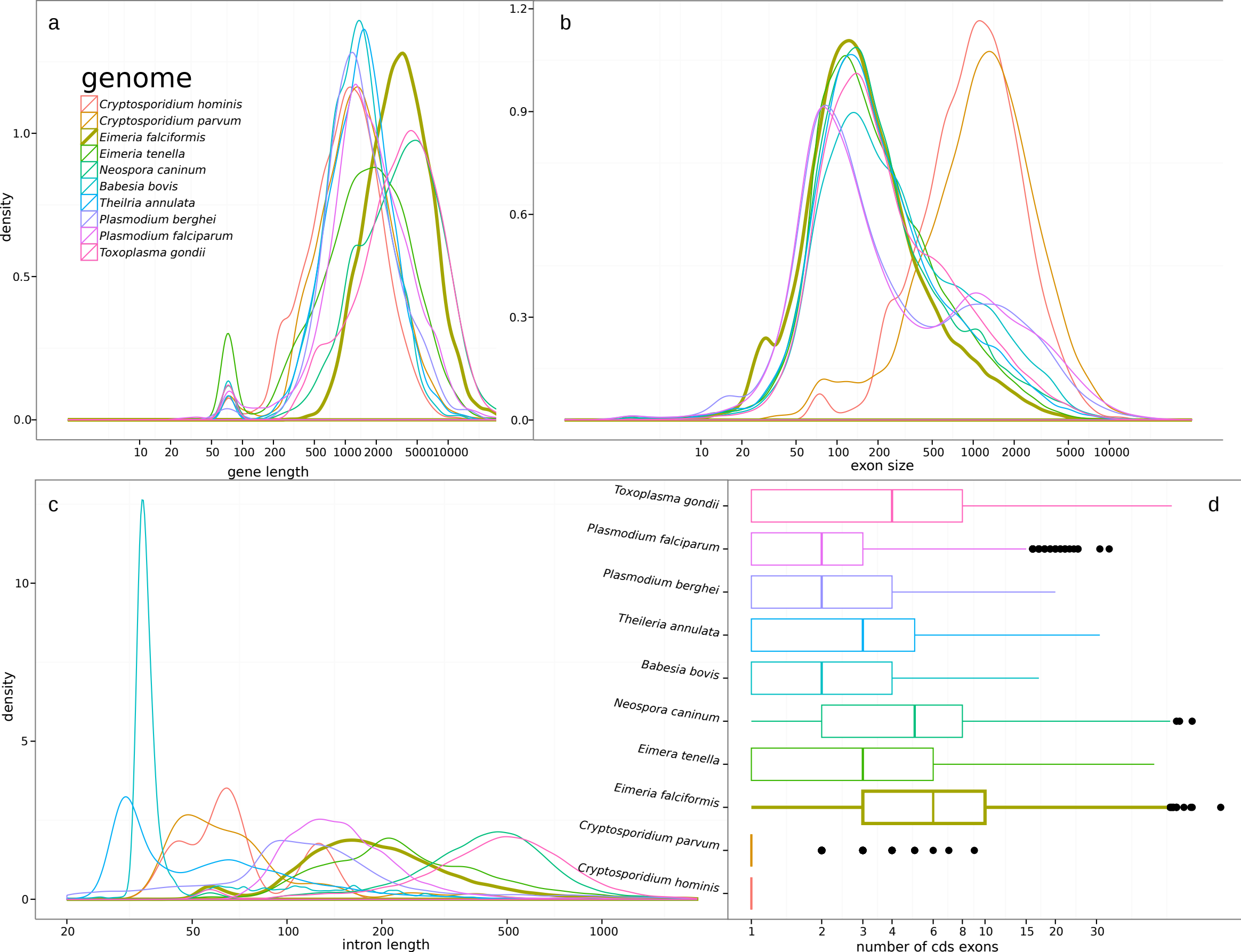

Supplement: Supplementary file 4 — Additional file 4: Structural comparison of protein coding genome features of Apicomplexa (figure). Protein coding features of apicomplexan genomes obtained from EupathDB (see Additional file 16 for exact versions) were compared with gene-predictions for E. falciformis obtained combining expression evidence (RNAseq) with ab initio gene-finding using Augustus [72]. Panels show the distribution of a) the overall length of genes, b) the size of individual exons and c) introns and d) the number of coding sequence exons per gene. (PDF 144 KB) [file 12864_2014_6777_MOESM4_ESM.pdf]

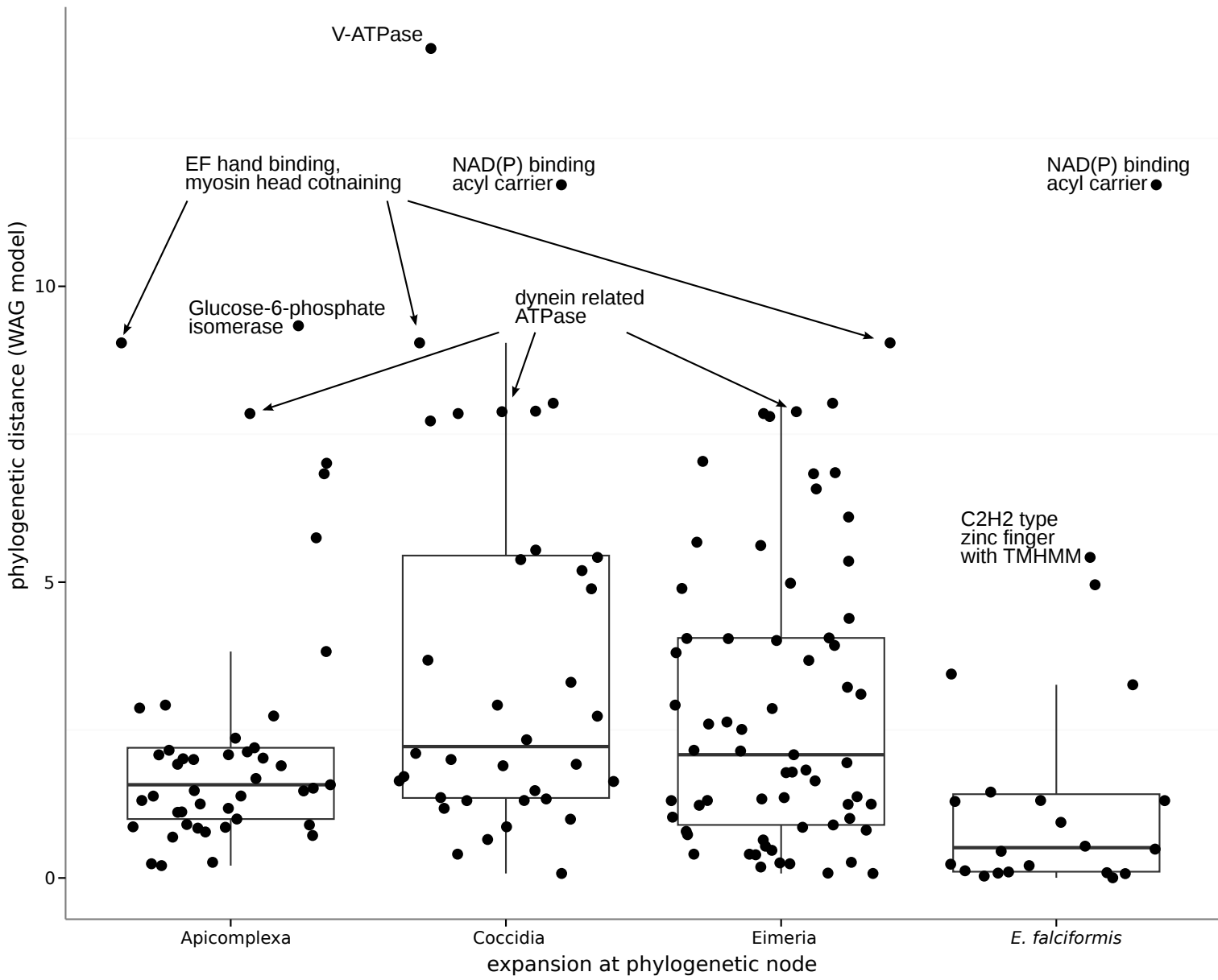

Supplement: Supplementary file 10 — Additional file 10: Pairwise phylogenetic distance genes expanded at different nodes of the apicomplexan phylogeny (figure). Boxplots (overlayed with single datapoints) are given for the the maximal phylogenetic distance of two genes in an expanded ortholog clusters as estimated by the WAG model in PhyML [72]. Ortholog clusters are grouped by expansions at different nodes of the apicomplexan phylogeny. (PDF 38 KB) [file 12864_2014_6777_MOESM10_ESM.pdf]

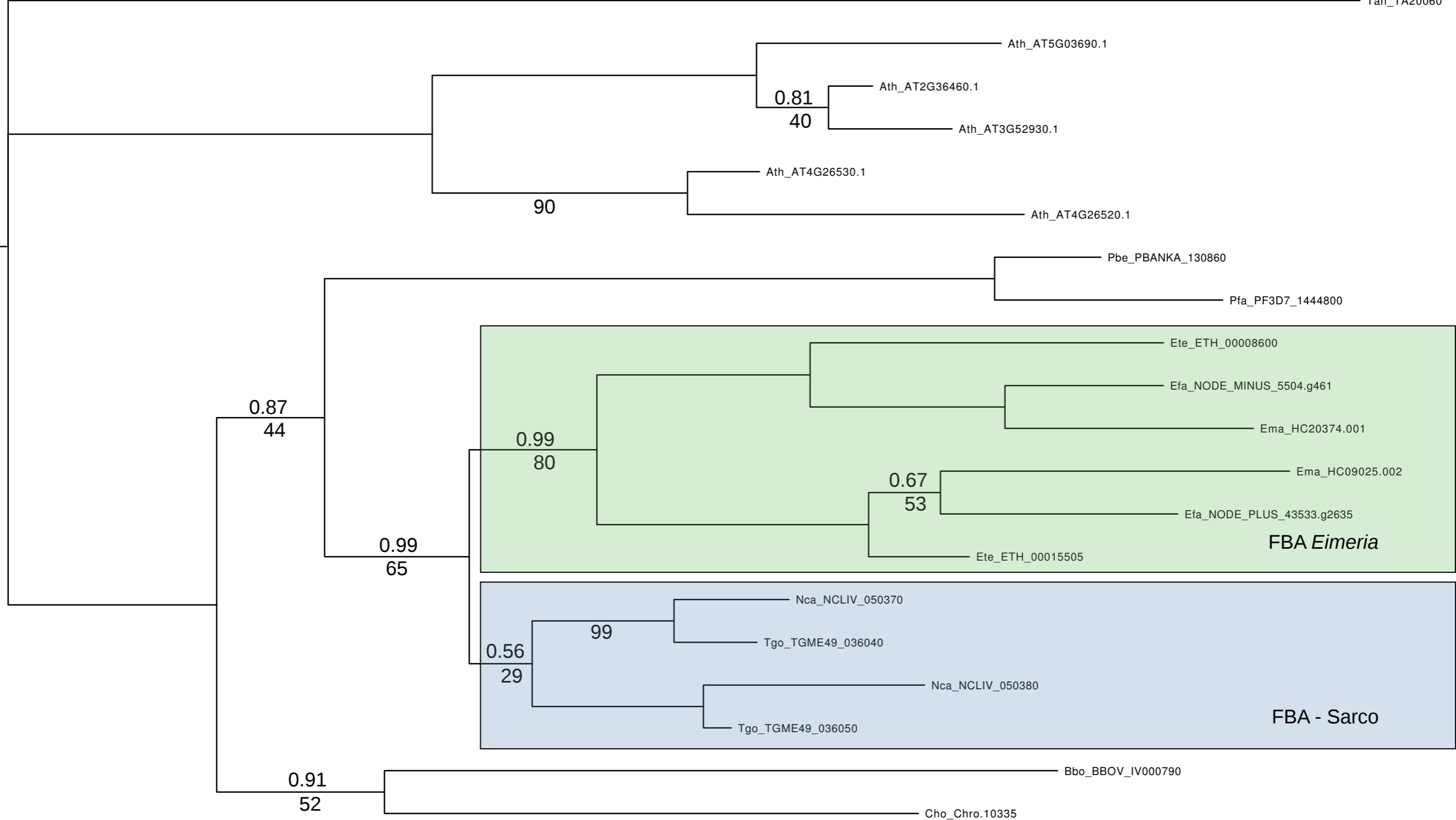

Supplement: Supplementary file 11 — Additional file 11: Phylogenetic tree for Fructose bisphosphate aldolase (figure). The ortholog cluster containing E. falciformis genes annotated as fructose bisphosphate aldolase (FBA) was found to be independently expanded in both Eimeriidae and Sarcocystidae. Sequences for the ortholog group were aligned using muscle, trimmed with trimAl and a phylogenetic tree was inferred with Mister Bayes and PhyML. Labels below branches give the number of bootstrap replicates supporting the clade (out of 100), labels above branches indicate its bayesian posterior probability. For branches with 100% bootstrap support or a posterior probability of 1, labels are omitted. After gene duplication in the Sarcocystidae (FBA Sarco), both paralogous copies were retained in T. gondii and N. caninum. The same fate was experienced by the independently duplicated genes in Eimeria (FBA Eimeria), E. tenella, E. falciformis and E. maxima each retained their paralogs. (PDF 32 KB) [file 12864_2014_6777_MOESM11_ESM.pdf]

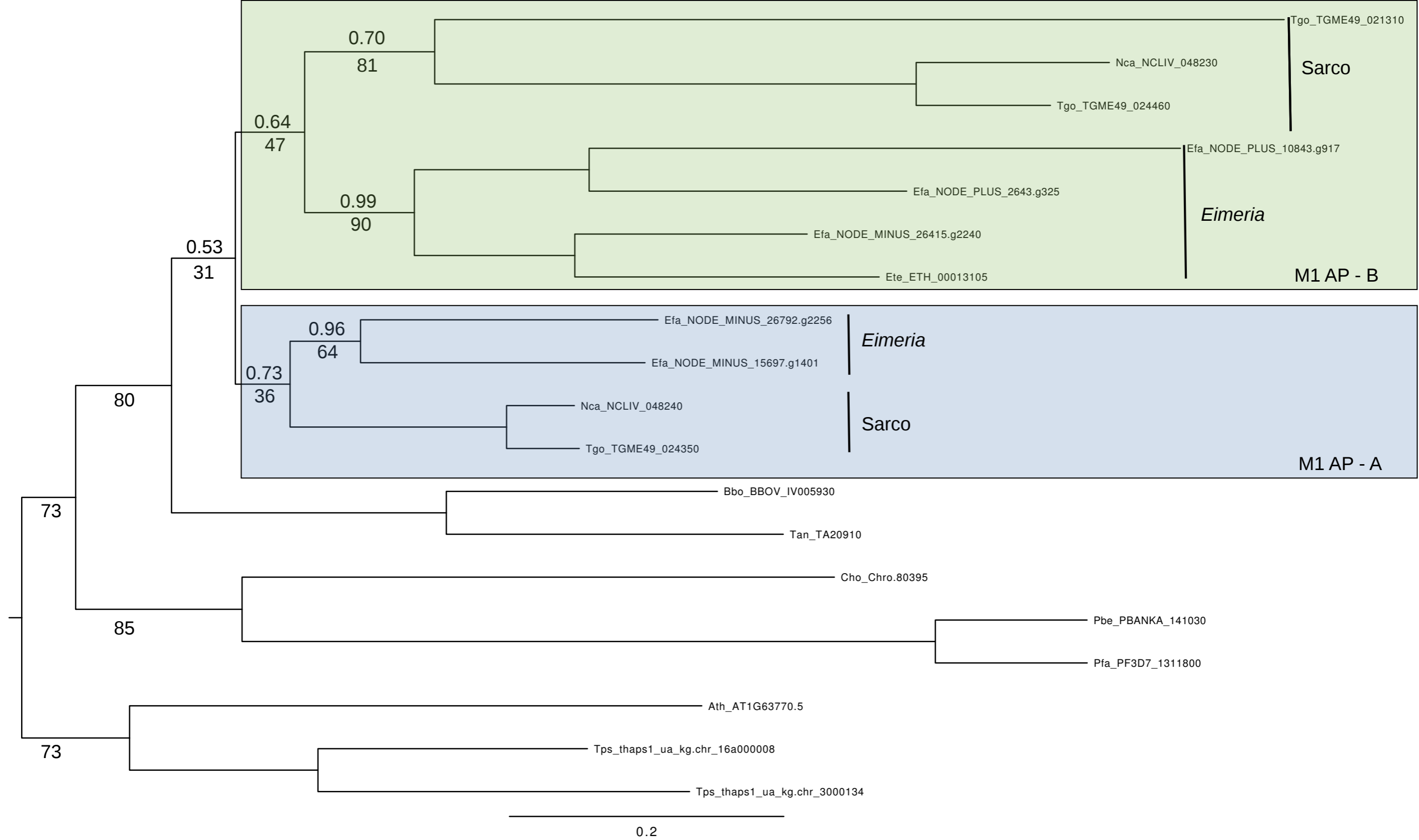

Supplement: Supplementary file 12 — Additional file 12: Phylogenetic tree for M1-amylopeptidase (figure). The ortholog cluster containing E. falciformis genes annotated as M1-amylopeptidase (M1 AP) was found to be independently expanded in both Eimeriidae and Sarcocystidae. Sequences for the ortholog group were aligned using muscle, trimmed with trimAl and a phylogenetic tree was inferred with Mister Bayes and PhyML. Labels below branches give the number of bootstrap replicates supporting the clade (out of 100), labels above branches indicate its bayesian posterior probability. For branches with 100% bootstrap support or a posterior probability of 1 labels are omitted. A first duplication common to the Coccidia, preceding the split of the Sarcocystidae and Eimeriidae created two copies of the gene. In one of the resulting subclades (M1AP -A) N. caninum and T. gondii genes were conserved in one copy, the E. tenella gene was lost and the E. falciformis gene duplicated. In the other subclade (M1AP - B) the gene was further duplicated after the split of the Sarcocystidae and Eimeriidae leaving two T. gondii paralogs after the loss of one ortholog in N. caninum. Similarly, in the Eimeriidae after initial duplication one of the paralogs was lost in E. tenella, and the E. falciformis paralog expanded in an additional duplication. (PDF 33 KB) [file 12864_2014_6777_MOESM12_ESM.pdf]

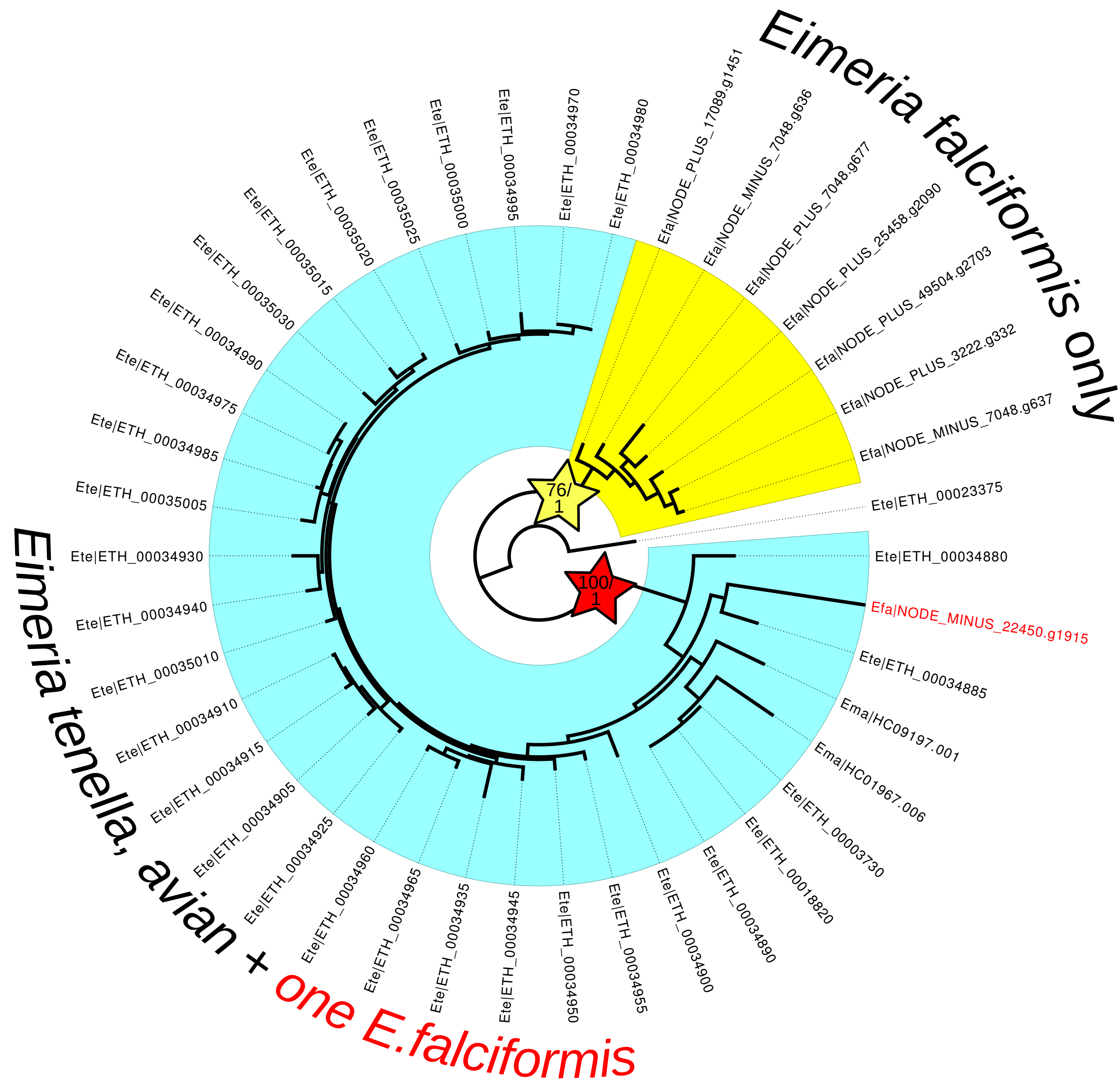

Supplement: Supplementary file 13 — Additional file 13: Independent expansion of surface antigens. Surface antigen domain (TA4) containing genes are enriched in both E. falciformis and E. tenella expanded gene families. Phylogenetic trees were constructed based a HMM guided alignment using MrBayes and PhyML and merged. The nodes separating avian and rodent SAG clades are highlighted with stars, within these supporting bootstrap replicates (from 100) and bayesian posterior probabilities are given. This tree confirms the independent expansion of two clades in E. falciformis and E. tenella and further highlights one E. falciformis gene with higher cross-species similarity to avian than rodent Eimeria. (PDF 35 KB) [file 12864_2014_6777_MOESM13_ESM.pdf]

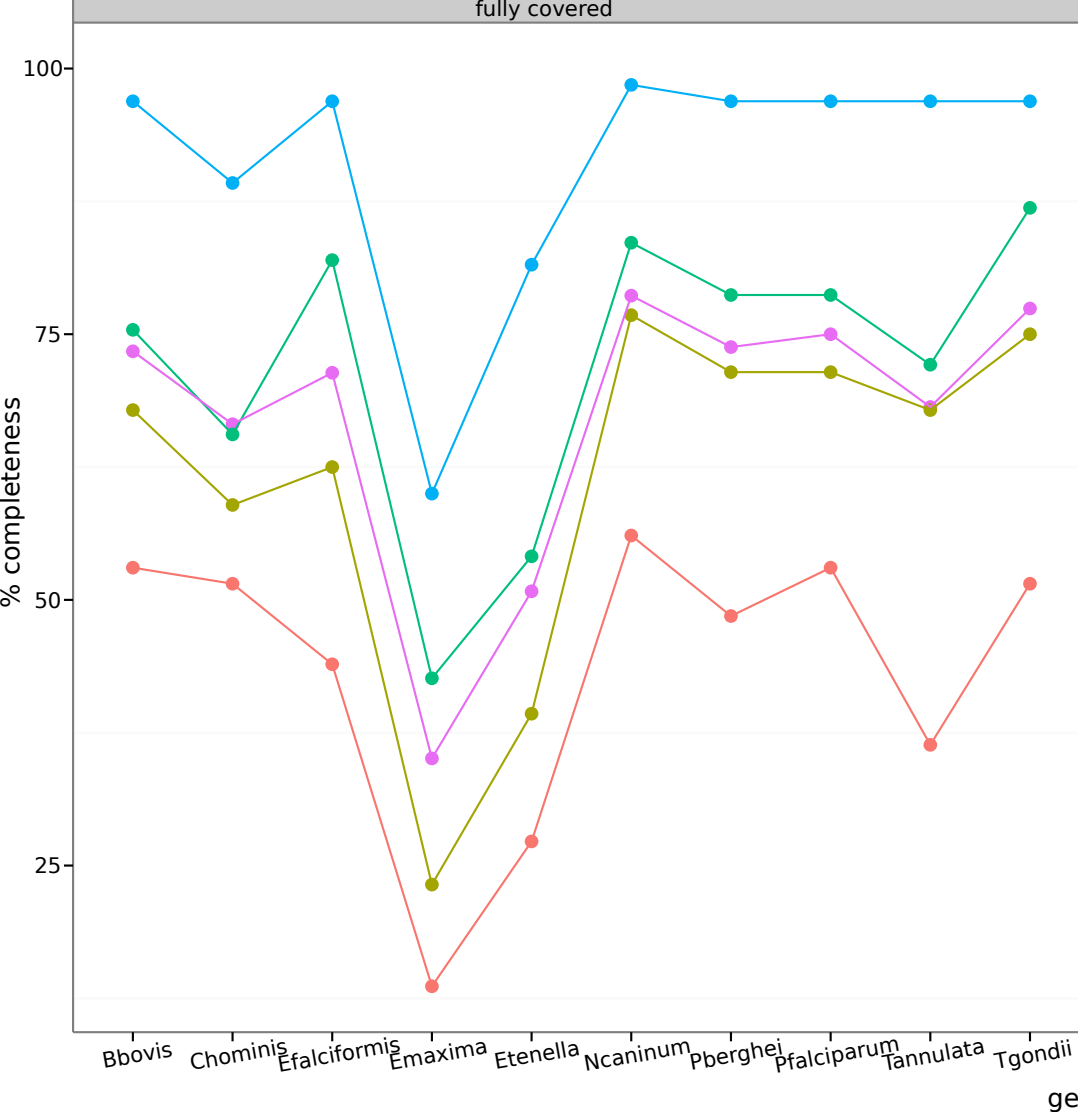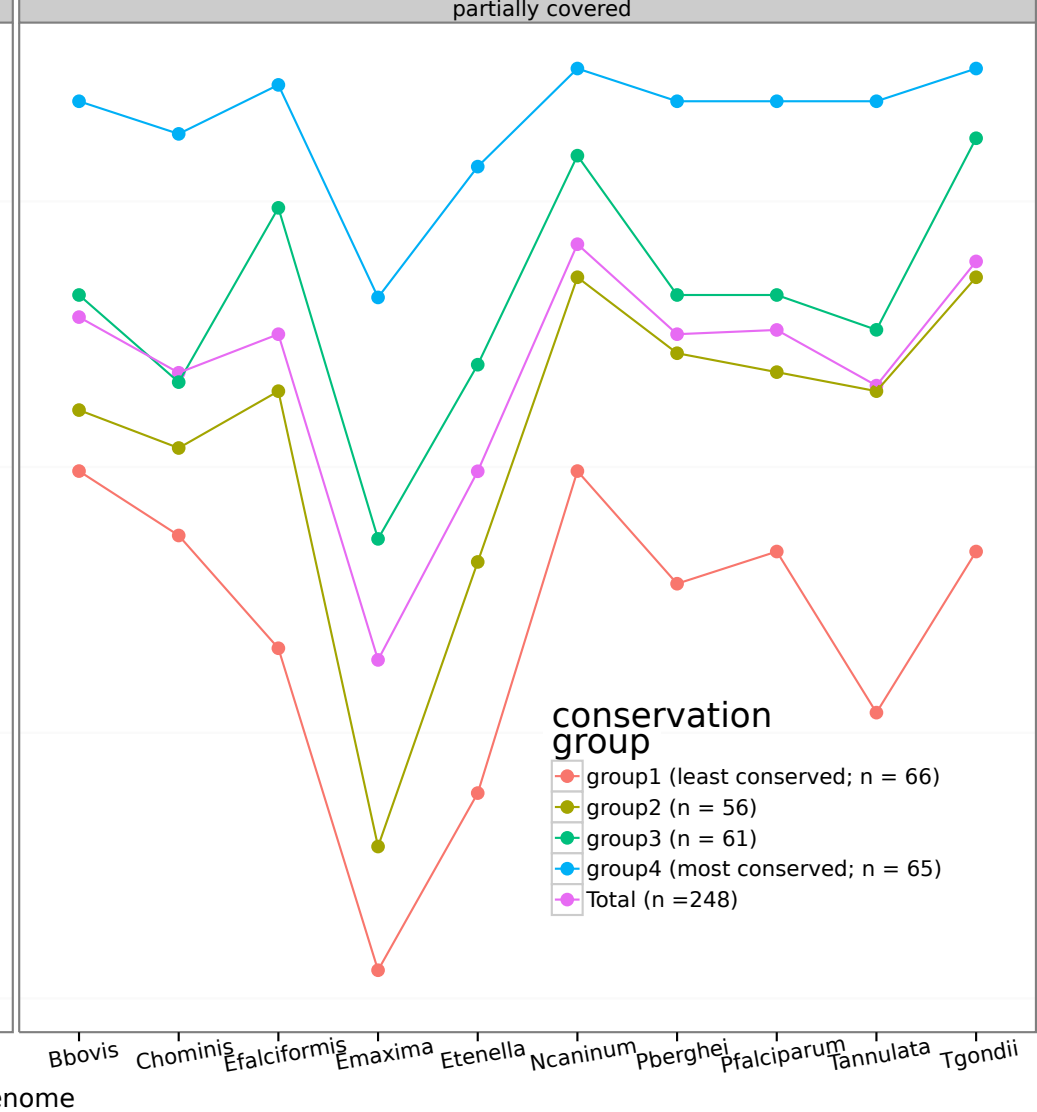

Supplement: Supplementary file 15 — Additional file 15: Evaluation of genome assembly completeness (figure). Apicomplexan genomes were searched for HMMs of Core Eukaryote Genes (CEGs) using CEGMA [61]. Parra et al. [79] divided these CEGs into four groups according to the degree of conservation observed in the pairwise alignments (4 being the most conserved group, 1 the least). Apicomplexan genome is general show a high divergence of CEGs visible in the low recovery of less conserved groups. CEGs for which proteins are longer than 70% of the corresponding HMM alignment are recognized as fully covered, others as partially covered. Group 4, expected to be fully present even in the reduced genomes of Apicomplexa is with 97% (63 genes) represented nearly completely in our assembly. The representation of less conserved groups 1–3 compares well with the high quality genomes of Plasmodium falciparum[80] and T. gondii[13]. (PDF 18 KB) [file 12864_2014_6777_MOESM15_ESM.pdf]
