# Supplementary material for: The genome of Eimeria falciformis - reduction and specialization in a single host apicomplexan parasite
Source: BMC Genomics. 2014 Aug 20;15(1):696. doi: 10.1186/1471-2164-15-696 (PMC4287421; doi:10.1186/1471-2164-15-696)
Supplement: Supplementary file 8 — Additional file 8: Enrichment analyses for ortholog clusters novel in certain clades of the apicomplexan phylogeny (figure containing tables). Gene sets identified as novel in certain clades of the apicomplexan phylogeny (Figure 3), were searched for enriched function using F-tests. The tables list enrichment results for GO molecular function, biological process, InterProScan domains and KEGG pathways. Table columns indicate IDs and names of the annotation, how often the annotation was found in the all ortholog clusterd E. falciformis genes, how many genes would be expected by chance based on this and the size of a gene-set and the p-value for the enrichment. (PDF 48 KB) [file 12864_2014_6777_MOESM8_ESM.pdf]

Id	description	Annotated	In group	Expected	p-value
GO:0001071	nucleic acid binding transcription factor activity	18	14	2.65	3.0e-09
GO:0003700	sequence-specific DNA binding transcription factor activity	18	14	2.65	3.0e-09
GO:0008270	zinc ion binding	162	40	23.81	3.4e-4
GO:0005515	protein binding	547	104	80.41	8.7e-3
GO:0046914	transition metal ion binding	181	40	26.61	0.003
GO:0016773	phosphotransferase activity, alcohol group as acceptor	132	31	19.40	0.003
GO:0004672	protein kinase activity	100	25	14.70	0.003
GO:0016301	kinase activity	135	31	19.84	0.005
GO:0005488	binding	1633	258	240.04	0.010
GO:0016772	transferase activity, transferring phosphorus-containing groups	211	43	31.02	0.011
GO:0006355	regulation of transcription, DNA-dependent	39	15	4.25	4.8e-06
GO:0043412	macromolecule modification	194	39	21.15	3.4e-05
GO:0010468	regulation of gene expression	45	15	4.91	3.6e-05
GO:0006464	cellular protein modification process	170	35	18.54	5.7e-05
IPR001471	AP2/ERF domain	17	14	1.919	3.0e-11
IPR021288	Sporozoite TA4 surface antigen	11	10	1.246	3.6e-09
IPR011009	Protein kinase-like domain	114	29	12.813	3.6e-05
IPR013083	Zinc finger, RING/FYVE/PHD-type	57	17	6.444	1.4e-04
IPR000571	Zinc finger, CCCH-type	20	9	2.270	1.7e-04
IPR000719	Protein kinase, catalytic domain	96	23	10.835	0.001
IPR002290	Serine/threonine- / dual-specificity protein kinase	86	21	9.715	0.001
IPR001611	Leucine-rich repeat	20	8	2.272	0.001
IPR002013	Synaptojanin, N-terminal	3	3	0.342	0.001
IPR016024	Armadillo-type fold	99	22	11.189	0.002
IPR003591	Leucine-rich repeat, typical subtype	6	4	0.683	0.002
IPR001841	Zinc finger, RING-type	32	10	3.633	0.002
IPR007087	Zinc finger, C2H2	14	6	1.593	0.003
IPR001965	Zinc finger, PHD-type	10	5	1.138	0.003
IPR019786	Zinc finger, PHD-type, conserved site	7	4	0.797	0.004
IPR011011	Zinc finger, FYVE/PHD-type	12	5	1.366	0.008
IPR011990	Tetratricopeptide-like helical	65	15	7.371	0.009
IPR002937	Amine oxidase	5	3	0.570	0.012
IPR004855	Transcription factor IIA, alpha/beta subunit	2	2	0.228	0.013
IPR013771	Bifunctional trypsin/alpha-amylase inhibitor helical domain	2	2	0.228	0.013
IPR019515	Vacuolar protein sorting-associated protein 54	2	2	0.228	0.013
IPR019562	Micronemal adhesive repeat, sialic-acid binding	2	2	0.228	0.013
IPR020635	Tyrosine-protein kinase, catalytic domain	2	2	0.228	0.013
IPR021869	Ribonuclease Zc3h12a-like	2	2	0.228	0.013
IPR019787	Zinc finger, PHD-finger	9	4	1.025	0.013
IPR020683	Ankyrin repeat-containing domain	26	7	2.959	0.023
IPR001357	BRCT domain	11	4	1.254	0.029
IPR011989	Armadillo-like helical	76	15	8.629	0.029
IPR001179	Peptidyl-prolyl cis-trans isomerase, FKBP-type, domain	3	2	0.342	0.036
IPR001778	Pollen allergen Poa pIX/Phl pVI	3	2	0.342	0.036
IPR003152	PIK-related kinase, FATC	3	2	0.342	0.036
IPR003653	Peptidase C48, SUMO/Sentrin/Ubl1	3	2	0.342	0.036
IPR005522	Inositol polyphosphate kinase	3	2	0.342	0.036
IPR017877	Myb-like domain	3	2	0.342	0.036
IPR000403	Phosphatidylinositol 3-/4-kinase, catalytic domain	7	3	0.798	0.037
IPR001752	Kinesin, motor domain	17	5	1.937	0.037
IPR002110	Ankyrin repeat	24	6	2.734	0.049
00513	Various types of N-glycan biosynthesis	7	2	0.218	0.021
00510	N-Glycan biosynthesis	9	2	0.281	0.034

Id	description	Annotated	In group	Expected	p-value
GO:0050515	4-(cytidine 5'-diphospho)-2-C-methyl-D-erythritol kinase	1	1	0	0.0013
GO:0006721	terpenoid metabolic process	4	1	0.01	0.0053
GO:0006720	isoprenoid metabolic process	7	1	0.01	0.0093
GO:0008610	lipid biosynthetic process	26	1	0.03	0.0345
IPR013200	HAD superfamily hydrolase-like, type 3	5	3	0.004	1.6e-08
IPR023214	HAD-like domain	39	3	0.036	1.4e-05
IPR004424	4-diphosphocytidyl-2C-methyl-D-erythritol kinase	1	1	0.001	0.001
IPR010909	PLAC	1	1	0.001	0.001
IPR006379	HAD-superfamily hydrolase, subfamily IIB	5	1	0.005	0.006
IPR014721	Ribosomal protein S5 domain 2-type fold, subgroup	16	1	0.018	0.020
IPR020568	Ribosomal protein S5 domain 2-type fold	25	1	0.029	0.032

Id	description	Annotated	In group	Expected	p-value
GO:0005509	calcium ion binding	66	21	7.30	3.3e-06
GO:0004112	cyclic-nucleotide phosphodiesterase	11	7	1.22	4.2e-05
GO:0008081	phosphoric diester hydrolase activity	16	8	1.77	0.00012
GO:0004252	serine-type endopeptidase activity	20	9	2.21	0.00012
GO:0008236	serine-type peptidase activity	26	10	2.87	0.00024
GO:0005515	protein binding	547	80	60.48	0.00185
GO:0042578	phosphoric ester hydrolase activity	44	12	4.87	0.00211
GO:0004609	phosphatidylserine decarboxylase	3	2	0.33	0.03388
GO:0044763	single-organism cellular process	387	60	40.12	0.00014
GO:0044699	single-organism process	417	62	43.23	0.00039
GO:0007165	signal transduction	62	15	6.43	0.00107
GO:0023052	signaling	62	15	6.43	0.00107
GO:0044700	single organism signaling	62	15	6.43	0.00107
GO:0065007	biological regulation	157	28	16.28	0.00178
GO:0007154	cell communication	65	15	6.74	0.00179
GO:0050794	regulation of cellular process	140	25	14.52	0.00317
GO:0051716	cellular response to stimulus	101	19	10.47	0.00568
GO:0050789	regulation of biological process	148	25	15.34	0.00685
IPR015500	Peptidase S8, subtilisin-related	9	7	0.824	1.7e-06
IPR000209	Peptidase S8/S53, subtilisin/kexin/sedolisin	10	7	0.915	5.1e-06
IPR023828	Peptidase S8, subtilisin, Ser-active site	8	6	0.733	1.4e-05
IPR002073	3'5'-cyclic nucleotide phosphodiesterase, catalytic domain	13	7	1.190	5.7e-05
IPR023088	3'5'-cyclic nucleotide phosphodiesterase	10	6	0.916	9.1e-05
IPR023827	Peptidase S8, subtilisin, Asp-active site	7	5	0.642	1.2e-04
IPR011992	EF-hand-like domain	64	16	5.830	1.7e-04
IPR006210	Epidermal growth factor-like	8	5	0.734	2.9e-04
IPR022398	Peptidase S8, subtilisin, His-active site	5	4	0.459	3.3e-04
IPR017986	WD40-repeat-containing domain	117	23	10.631	0.001
IPR023393	START-like domain	9	5	0.826	0.001
IPR015943	WD40/YVTN repeat-like-containing domain	121	23	10.999	0.001
IPR001680	WD40 repeat	100	20	9.102	0.001
IPR000152	EGF-type aspartate/asparagine hydroxylation site	6	4	0.551	0.001
IPR001881	EGF-like calcium-binding	6	4	0.551	0.001
IPR023174	3'5'-cyclic nucleotide phosphodiesterase, conserved site	10	5	0.917	0.001
IPR018249	EF-HAND 2	53	12	4.845	0.003
IPR003607	HD/PDEase domain	9	4	0.827	0.006
IPR001810	F-box domain, cyclin-like	5	3	0.460	0.007
IPR018097	EGF-like calcium-binding, conserved site	5	3	0.460	0.007
IPR002048	Calcium-binding EF-hand	32	8	2.932	0.007
IPR000172	Glucose-methanol-choline oxidoreductase, N-terminal	2	2	0.184	0.008
IPR004326	Mlo-related protein	2	2	0.184	0.008
IPR005221	Phosphatidylserine decarboxylase	2	2	0.184	0.008
IPR007751	Domain of unknown function DUF676, lipase-like	2	2	0.184	0.008
IPR007867	Glucose-methanol-choline oxidoreductase, C-terminal	2	2	0.184	0.008
IPR000884	Thrombospondin, type 1 repeat	15	5	1.377	0.009
IPR001214	SET domain	15	5	1.377	0.009
IPR009030	Growth factor, receptor	17	5	1.561	0.016
IPR000133	ER lumen protein retaining receptor	3	2	0.276	0.024
IPR000782	FAS1 domain	3	2	0.276	0.024
IPR000800	Notch domain	3	2	0.276	0.024
IPR000845	Nucleoside phosphorylase domain	3	2	0.276	0.024
IPR003817	Phosphatidylserine decarboxylase-related	3	2	0.276	0.024
IPR023395	Mitochondrial carrier domain	19	5	1.745	0.026
IPR018488	Cyclic nucleotide-binding, conserved site	8	3	0.736	0.031
IPR014710	RmlC-like jelly roll fold	14	4	1.286	0.034
IPR011990	Tetratricopeptide-like helical	71	12	6.503	0.036
IPR000595	Cyclic nucleotide-binding domain	9	3	0.828	0.043
IPR000742	Epidermal growth factor-like domain	9	3	0.828	0.043
IPR018490	Cyclic nucleotide-binding-like	9	3	0.828	0.043
IPR000436	Sushi/SCR/CCP	4	2	0.368	0.045
IPR002913	Lipid-binding START	4	2	0.368	0.045
IPR003298	Apical membrane antigen 1	4	2	0.368	0.045
IPR019775	WD40 repeat, conserved site	50	9	4.585	0.045
00563	Glycosylphosphatidylinositol(GPI)-anchor biosynthesis	8	2	0.226	0.023
00512	Mucin type O-glycan biosynthesis	1	1	0.029	0.031
00311	Penicillin and cephalosporin biosynthesis	1	1	0.029	0.031
00230	Purine metabolism	61	5	1.572	0.032

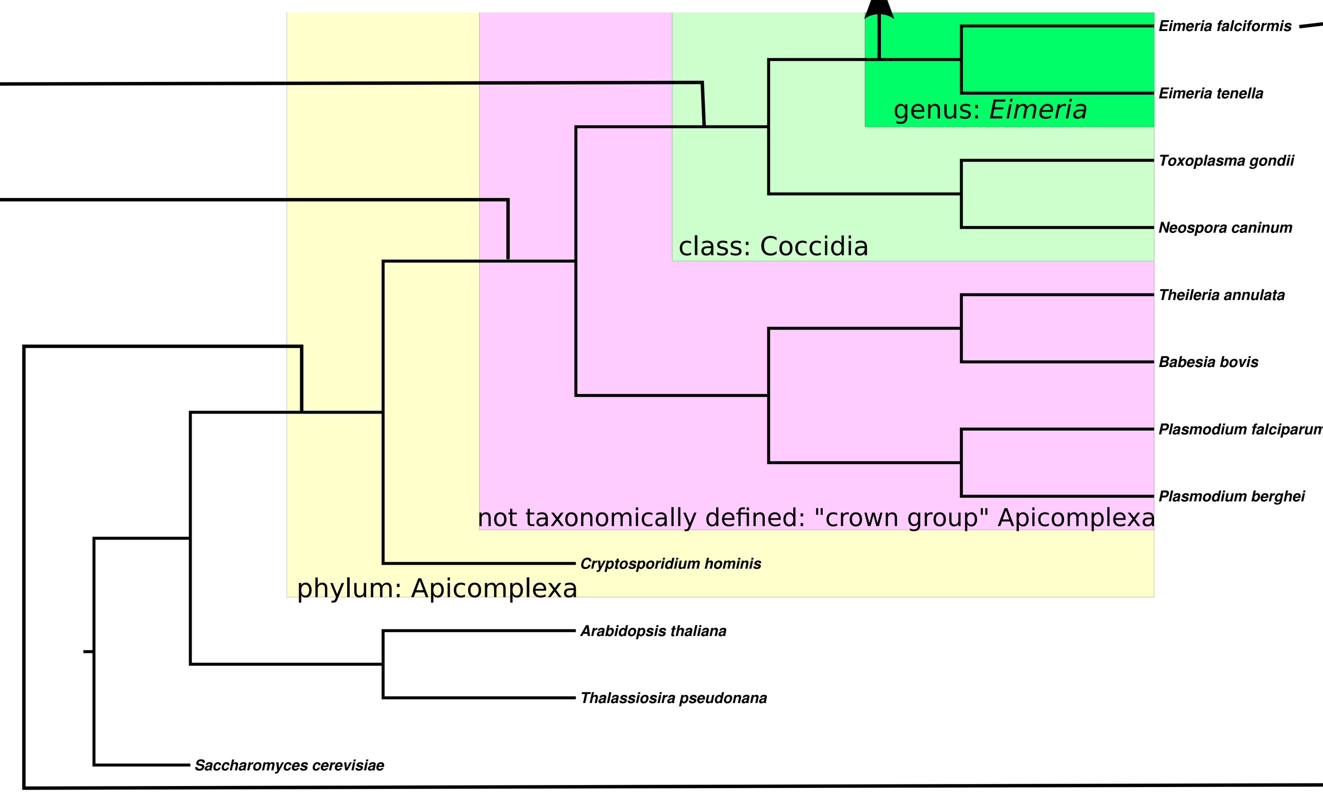

Id	description	Annotated	In group	Expected	p-value
GO:0005515	protein binding	547	53	36.72	0.0014
GO:0060589	nucleoside-triphosphatase regulator activity	22	6	1.48	0.0025
GO:0043169	cation binding	287	31	19.27	0.0036
GO:0008047	enzyme activator activity	17	5	1.14	0.0041
GO:0005044	scavenger receptor activity	2	2	0.13	0.0045
GO:0038024	cargo receptor activity	2	2	0.13	0.0045
GO:0048038	quinone binding	2	2	0.13	0.0045
GO:0046872	metal ion binding	279	30	18.73	0.0046
GO:0004872	receptor activity	6	3	0.40	0.0051
GO:0005083	small GTPase regulator activity	19	5	1.28	0.0069
GO:0007154	cell communication	65	16	3.83	3.3e-07
GO:0007165	signal transduction	62	15	3.65	1.1e-06
GO:0023052	signaling	62	15	3.65	1.1e-06
GO:0044700	single organism signaling	62	15	3.65	1.1e-06
GO:0044699	single-organism process	417	42	24.55	3.5e-05
GO:0050794	regulation of cellular process	140	20	8.24	8.7e-05
GO:0035556	intracellular signal transduction	42	10	2.47	9.3e-05
GO:0051716	cellular response to stimulus	101	16	5.95	0.00014
GO:0050789	regulation of biological process	148	20	8.71	0.00019
GO:0044763	single-organism cellular process	387	38	22.78	0.00021
IPR004043	LCCL	4	4	0.269	2.1e-05
IPR008973	C2 calcium/lipid-binding domain, CaLB	7	5	0.471	2.6e-05
IPR000008	C2 calcium-dependent membrane targeting	6	4	0.404	2.8e-04
IPR000772	Ricin B lectin domain	6	4	0.404	2.8e-04
IPR018029	C2 membrane targeting protein	6	4	0.404	2.8e-04
IPR021691	Protein of unknown function DUF3273	3	3	0.202	3.1e-04
IPR008979	Galactose-binding domain-like	7	4	0.471	0.001
IPR022086	Inner membrane complex protein	8	4	0.539	0.001
IPR001251	CRAL-TRIO domain	9	4	0.606	0.002
IPR011074	CRAL/TRIO, N-terminal domain	5	3	0.337	0.003
IPR002110	Ankyrin repeat	24	6	1.614	0.004
IPR001190	Speract/scavenger receptor	2	2	0.135	0.005
IPR002181	Fibrinogen, alpha/beta/gamma chain, C-terminal globular	2	2	0.135	0.005
IPR003104	Actin-binding FH2/DRF autoregulatory	2	2	0.135	0.005
IPR003131	Potassium channel tetramerisation-type BTB domain	2	2	0.135	0.005
IPR006685	Mechanosensitive ion channel MscS	2	2	0.135	0.005
IPR015801	Copper amine oxidase, N2/N3-terminal	2	2	0.135	0.005
IPR017448	Speract/scavenger receptor-related	2	2	0.135	0.005
IPR019385	Phosphorylated adapter RNA export protein, RNA-binding domain	2	2	0.135	0.005
IPR020683	Ankyrin repeat-containing domain	26	6	1.749	0.007
IPR011333	BTB/POZ fold	7	3	0.472	0.009
IPR000315	Zinc finger, B-box	3	2	0.203	0.013
IPR000421	Coagulation factor 5/8 C-terminal type domain	3	2	0.203	0.013
IPR000756	Diacylglycerol kinase, accessory domain	3	2	0.203	0.013
IPR001173	Glycosyl transferase, family 2	3	2	0.203	0.013
IPR001206	Diacylglycerol kinase, catalytic domain	3	2	0.203	0.013
IPR011658	PA14	3	2	0.203	0.013
IPR015425	Actin-binding FH2	3	2	0.203	0.013
IPR004087	K Homology domain	8	3	0.540	0.013
IPR013320	Concanavalin A-like lectin/glucanase, subgroup	8	3	0.540	0.013
IPR023393	START-like domain	8	3	0.540	0.013
IPR013083	Zinc finger, RING/FYVE/PHD-type	57	9	3.827	0.014
IPR023214	HAD-like domain	39	7	2.622	0.015
IPR008985	Concanavalin A-like lectin/glucanase	15	4	1.011	0.016
IPR001841	Zinc finger, RING-type	32	6	2.154	0.019
IPR004088	K Homology domain, type 1	9	3	0.607	0.019
IPR001401	Dynamin, GTPase domain	4	2	0.270	0.025
IPR002913	Lipid-binding START	4	2	0.270	0.025
IPR011016	Zinc finger, RING-CH-type	4	2	0.270	0.025
IPR013714	Golgi apparatus membrane protein TVP15	4	2	0.270	0.025
IPR011701	Major facilitator superfamily	18	4	1.214	0.030
IPR000195	Rab-GTPase-TBC domain	11	3	0.742	0.034
IPR013783	Immunoglobulin-like fold	11	3	0.742	0.034
IPR000169	Cysteine peptidase, cysteine active site	5	2	0.338	0.040
IPR000225	Armadillo	5	2	0.338	0.040
IPR000668	Peptidase C1A, papain C-terminal	5	2	0.338	0.040
IPR025660	Cysteine peptidase, histidine active site	5	2	0.338	0.040
00740	Riboflavin metabolism	2	1	0.035	0.038

Id	description	Annotated	In group	Expected	p-value
GO:0005216	ion channel activity	7	4	0.45	0.00051
GO:0015267	channel activity	7	4	0.45	0.00051
GO:0022803	passive transmembrane transporter activity	7	4	0.45	0.00051
GO:0022838	substrate-specific channel activity	7	4	0.45	0.00051
GO:0003735	structural constituent of ribosome	100	15	6.49	0.00155
GO:0022836	gated channel activity	5	3	0.32	0.00244
GO:0022839	ion gated channel activity	5	3	0.32	0.00244
GO:0015075	ion transmembrane transporter activity	56	10	3.64	0.00262
GO:0005198	structural molecule activity	121	16	7.86	0.00413
GO:0004129	cytochrome-c oxidase activity	2	2	0.13	0.00419
GO:0044267	cellular protein metabolic process	429	38	25.83	0.0032
GO:0006813	potassium ion transport	2	2	0.12	0.0036
GO:0006412	translation	169	19	10.17	0.0040
GO:0010467	gene expression	311	28	18.72	0.0117
GO:0019538	protein metabolic process	516	41	31.06	0.0167
GO:0009405	pathogenesis	5	2	0.30	0.0318
GO:0051704	multi-organism process	5	2	0.30	0.0318
GO:0044260	cellular macromolecule metabolic process	675	49	40.64	0.0432
GO:0006397	mRNA processing	14	3	0.84	0.0474
GO:0032259	methylation	14	3	0.84	0.0474
IPR006387	CPW-WPC domain	5	4	0.291	5.6e-05
IPR011641	Tyrosine-protein kinase ephrin type A/B receptor-like	3	3	0.175	2.0e-04
IPR012677	Nucleotide-binding, alpha-beta plait	65	12	3.747	3.3e-04
IPR005135	Endonuclease/exonuclease/phosphatase	8	4	0.466	0.001
IPR013584	RAP domain	9	4	0.524	0.001
IPR000504	RNA recognition motif domain	55	10	3.180	0.001
IPR000001	Kringle	2	2	0.117	0.003
IPR002483	Splicing factor PWI	2	2	0.117	0.003
IPR003929	Potassium channel, calcium-activated, BK, alpha subunit	2	2	0.117	0.003
IPR007621	TPM domain	2	2	0.117	0.003
IPR010441	Protein of unknown function DUF1042	2	2	0.117	0.003
IPR013099	Ion transport 2	2	2	0.117	0.003
IPR013806	Kringle-like fold	2	2	0.117	0.003
IPR021150	Ubiquinol-cytochrome c chaperone/UPF0174	2	2	0.117	0.003
IPR025677	OST-HTH associated domain	2	2	0.117	0.003
IPR001461	Peptidase A1	6	3	0.350	0.004
IPR009007	Peptidase aspartic, catalytic	6	3	0.350	0.004
IPR003409	MORN motif	12	4	0.699	0.004
IPR021109	Peptidase aspartic	8	3	0.467	0.009
IPR009069	MTCP1	3	2	0.175	0.010
IPR002877	Ribosomal RNA methyltransferase FtsJ domain	4	2	0.234	0.019
IPR003298	Apical membrane antigen 1	4	2	0.234	0.019
IPR008907	P25-alpha	4	2	0.234	0.019
IPR009543	Vacuolar protein sorting-associated protein	4	2	0.234	0.019
IPR012295	Beta2-adaptin/TBP, C-terminal domain	4	2	0.234	0.019
IPR023115	Translation initiation factor IF- 2, domain 3	4	2	0.234	0.019
IPR001849	Pleckstrin homology domain	5	2	0.292	0.030
IPR018355	SPla/RYanodine receptor subgroup	6	2	0.350	0.044
00240	Pyrimidine metabolism	52	3	0.413	0.018
