# Supplementary material for: The genome of Eimeria falciformis - reduction and specialization in a single host apicomplexan parasite
Source: BMC Genomics. 2014 Aug 20;15(1):696. doi: 10.1186/1471-2164-15-696 (PMC4287421; doi:10.1186/1471-2164-15-696)
Supplement: Supplementary file 9 — Additional file 9: Enrichment analyses for ortholog clusters expanded in certain clades of the apicomplexan phylogeny (figure containing tables). Genes sets contained in ortholog clusters expanded at certain clades of the apicomplexan phylogeny (Figure 3), were searched for enriched function using F-tests. The tables list enrichment results for GO molecular function, biological process, InterProScan domains and KEGG pathways. Table columns indicate IDs and names of the annotation, how often the annotation was found in the all gene/species-tree reconciliated E. falciformis genes, how many genes would be expected by chance based on this and the size of a gene-set and the p-value for the enrichment. (PDF 66 KB) [file 12864_2014_6777_MOESM9_ESM.pdf]

Expansion in *Eimeria falciformis*

Id	description	Annotated	In group	Expected	p-value
GO:0008237	metallopeptidase activity	23	8	0.36	5.5e-10
GO:0031177	phosphopantetheine binding	3	3	0.05	3.5e-06
GO:0072341	modified amino acid binding	3	3	0.05	3.5e-06
GO:0070011	peptidase activity, acting on L-amino acid peptides	75	8	1.19	1.1e-05
GO:0016597	amino acid binding	4	3	0.06	1.4e-05
GO:0008233	peptidase activity	80	8	1.26	1.8e-05
GO:0033218	amide binding	5	3	0.08	3.5e-05
GO:0008270	zinc ion binding	118	9	1.87	4.5e-05
GO:0019842	vitamin binding	7	3	0.11	0.00012
GO:0031406	carboxylic acid binding	7	3	0.11	0.00012
GO:0016567	protein ubiquitination	5	2	0.08	0.0023
GO:0032446	protein modification by small protein conjugation	6	2	0.09	0.0033
GO:0070647	protein modification by small protein conjugation or removal	6	2	0.09	0.0033
GO:0006508	proteolysis	81	5	1.27	0.0064
GO:0008654	phospholipid biosynthetic process	9	2	0.14	0.0078
GO:0006644	phospholipid metabolic process	14	2	0.22	0.0188
GO:0006511	ubiquitin-dependent protein catabolic process	20	2	0.31	0.0372
GO:0019941	modification-dependent protein catabolic process	20	2	0.31	0.0372
GO:0043632	modification-dependent macromolecule catabolic process	20	2	0.31	0.0372
GO:0008610	lipid biosynthetic process	23	2	0.36	0.0482
IPR021288	Sporozoite TA4 surface antigen	9	7	0.177	4.8e-11
IPR012779	Peptidase M1, alanyl aminopeptidase	5	5	0.100	3.5e-09
IPR014782	Peptidase M1, membrane alanine aminopeptidase, N-terminal	5	5	0.100	3.5e-09
IPR024601	Peptidase M1, alanyl aminopeptidase, C-terminal	5	5	0.100	3.5e-09
IPR009081	Acyl carrier protein-like	4	4	0.080	1.7e-07
IPR014031	Beta-ketoacyl synthase, C-terminal	5	4	0.100	8.5e-07
IPR006162	Phosphopantetheine attachment site	3	3	0.061	8.6e-06
IPR013578	Peptidase M16C associated	3	3	0.061	8.6e-06
IPR020806	Polyketide synthase, phosphopantetheine-binding domain	3	3	0.061	8.6e-06
IPR016038	Thiolase-like, subgroup	8	4	0.161	1.1e-05
IPR016039	Thiolase-like	8	4	0.161	1.1e-05
IPR001227	Acyl transferase domain	4	3	0.081	3.4e-05
IPR014030	Beta-ketoacyl synthase, N-terminal	4	3	0.081	3.4e-05
IPR014043	Acyl transferase	4	3	0.081	3.4e-05
IPR016036	Malonyl-CoA ACP transacylase, ACP-binding	4	3	0.081	3.4e-05
IPR020801	Polyketide synthase, acyl transferase domain	4	3	0.081	3.4e-05
IPR020841	Polyketide synthase, beta-ketoacyl synthase domain	4	3	0.081	3.4e-05
IPR020842	Polyketide synthase/Fatty acid synthase, KR	5	3	0.101	8.3e-05
IPR005221	Phosphatidylserine decarboxylase	2	2	0.041	4.2e-04
IPR013968	Polyketide synthase, KR	2	2	0.041	4.2e-04
IPR019474	Ubiquitin conjugation factor E4, core	2	2	0.041	4.2e-04
IPR020471	Aldo/keto reductase subgroup	2	2	0.041	4.2e-04
IPR020807	Polyketide synthase, dehydratase domain	2	2	0.041	4.2e-04
IPR016035	Acyl transferase/acyl hydrolase/lysophospholipase	8	3	0.162	0.0014
IPR016040	NAD(P)-binding domain	52	6	1.037	0.001
IPR002198	Short-chain dehydrogenase/reductase SDR	9	3	0.182	0.001
IPR007863	Peptidase M16, C-terminal	10	3	0.203	0.001
IPR011249	Metalloenzyme, LuxS/M16 peptidase-like, metal-binding	10	3	0.203	0.001
IPR003817	Phosphatidylserine decarboxylase-related	3	2	0.061	0.001
IPR006634	TRAM/LAG1/CLN8 homology domain	3	2	0.061	0.001
IPR013120	Male sterility, NAD-binding	3	2	0.061	0.001
IPR018201	Beta-ketoacyl synthase, active site	3	2	0.061	0.001
IPR005045	Protein of unknown function DUF284, transmembrane eukaryotic	4	2	0.082	0.002
IPR023174	3'5'-cyclic nucleotide phosphodiesterase, conserved site	5	2	0.102	0.004
IPR023210	NADP-dependent oxidoreductase domain	5	2	0.102	0.004
IPR001357	BRCT domain	6	2	0.122	0.006
IPR002073	3'5'-cyclic nucleotide phosphodiesterase, catalytic domain	6	2	0.122	0.006
IPR004274	NLI interacting factor	6	2	0.122	0.006
IPR023088	3'5'-cyclic nucleotide phosphodiesterase	6	2	0.122	0.006
IPR011237	Peptidase M16, core	8	2	0.163	0.011
IPR002347	Glucose/ribitol dehydrogenase	9	2	0.184	0.014
IPR000863	Sulfotransferase domain	1	1	0.021	0.021
IPR018170	Aldo/keto reductase, conserved site	1	1	0.021	0.021
00590	Arachidonic acid metabolism	3	2	0.035	0.001
00480	Glutathione metabolism	13	2	0.155	0.014
00564	Glycerophospholipid metabolism	22	2	0.267	0.038
00980	Metabolism of xenobiotics by cytochrome P450	5	2	0.059	0.002

Expansion in the genus *Eimeria*

Id	description	Annotated	In group	Expected	p-value
GO:0003774	motor activity	31	22	2.57	3.9e-18
GO:0017111	nucleoside-triphosphatase activity	215	47	17.85	1.8e-11
GO:0016462	pyrophosphatase activity	218	47	18.09	3.0e-11
GO:0016818	hydrolase activity, acting on phosphorus-containing anhydrides	219	47	18.18	3.6e-11
GO:0016817	hydrolase activity, acting on acid anhydrides	222	47	18.43	6.1e-11
GO:0016787	hydrolase activity	413	68	34.28	1.3e-10
GO:0003777	microtubule motor activity	22	13	1.83	1.4e-09
GO:0008237	metallopeptidase activity	23	9	1.91	4.4e-05
GO:0008061	chitin binding	4	4	0.33	4.6e-05
GO:0097367	carbohydrate derivative binding	4	4	0.33	4.6e-05
GO:0007017	microtubule-based process	34	18	2.54	8.6e-13
GO:0006928	cellular component movement	27	16	2.02	1.8e-12
GO:0007018	microtubule-based movement	27	16	2.02	1.8e-12
GO:0006022	aminoglycan metabolic process	4	4	0.30	2.9e-05
GO:0006030	chitin metabolic process	4	4	0.30	2.9e-05
GO:0006040	amino sugar metabolic process	5	4	0.37	0.00014
GO:1901071	glucosamine-containing compound metabolic process	5	4	0.37	0.00014
GO:0006461	protein complex assembly	10	4	0.75	0.00435
GO:0070271	protein complex biogenesis	10	4	0.75	0.00435
GO:0007596	blood coagulation	2	2	0.15	0.00554
IPR004273	Dynein heavy chain	10	10	0.843	2.0e-11
IPR013602	Dynein heavy chain, domain-2	10	10	0.843	2.0e-11
IPR024743	Dynein heavy chain, coiled coil stalk	10	10	0.843	2.0e-11
IPR001609	Myosin head, motor domain	9	9	0.760	2.3e-10
IPR024317	Dynein heavy chain, P-loop containing D4 domain	9	9	0.760	2.3e-10
IPR011704	ATPase, dynein-related, AAA domain	8	7	0.678	2.4e-07
IPR003593	AAA+ ATPase domain	64	18	5.364	4.0e-06
IPR012779	Peptidase M1, alanyl aminopeptidase	5	5	0.425	4.5e-06
IPR013594	Dynein heavy chain, domain-1	5	5	0.425	4.5e-06
IPR014782	Peptidase M1, membrane alanine aminopeptidase, N-terminal	5	5	0.425	4.5e-06
IPR024601	Peptidase M1, alanyl aminopeptidase, C-terminal	5	5	0.425	4.5e-06
IPR009030	Growth factor, receptor	11	7	0.932	7.9e-06
IPR002557	Chitin binding domain	4	4	0.340	5.3e-05
IPR007120	DNA-directed RNA polymerase, subunit 2, domain 6	3	3	0.256	0.001
IPR007121	RNA polymerase, beta subunit, conserved site	3	3	0.256	0.001
IPR007641	RNA polymerase Rpb2, domain 7	3	3	0.256	0.001
IPR007644	RNA polymerase, beta subunit, protrusion	3	3	0.256	0.001
IPR007645	RNA polymerase Rpb2, domain 3	3	3	0.256	0.001
IPR011641	Tyrosine-protein kinase ephrin type A/B receptor-like	3	3	0.256	0.001
IPR013578	Peptidase M16C associated	3	3	0.256	0.001
IPR017871	ABC transporter, conserved site	14	6	1.189	0.001
IPR014756	Immunoglobulin E-set	11	5	0.935	0.001
IPR000048	IQ motif, EF-hand binding site	4	3	0.341	0.002
IPR018316	Tubulin/FtsZ, 2-layer sandwich domain	4	3	0.341	0.002
IPR013781	Glycoside hydrolase, catalytic domain	8	4	0.681	0.003
IPR003439	ABC transporter-like	18	6	1.530	0.003
IPR001140	ABC transporter, transmembrane domain	9	4	0.766	0.005
IPR011527	ABC transporter, transmembrane domain, type 1	9	4	0.766	0.005
IPR017940	ABC transporter, integral membrane type 1	9	4	0.766	0.005
IPR008280	Tubulin/FtsZ, C-terminal	5	3	0.426	0.005
IPR013093	ATPase, AAA-2	5	3	0.426	0.005
IPR019489	Clp ATPase, C-terminal	5	3	0.426	0.005
IPR023123	Tubulin, C-terminal	5	3	0.426	0.005
IPR000001	Kringle	2	2	0.171	0.007
IPR000177	Apple domain	2	2	0.171	0.007
IPR000741	Fructose-bisphosphate aldolase, class-I	2	2	0.171	0.007
IPR001190	Speract/scavenger receptor	2	2	0.171	0.007
IPR002328	Alcohol dehydrogenase, zinc-type, conserved site	2	2	0.171	0.007
IPR002453	Beta tubulin	2	2	0.171	0.007
IPR002498	Phosphatidylinositol-4-phosphate 5-kinase, core	2	2	0.171	0.007
IPR004882	LUC7-related	2	2	0.171	0.007
IPR006650	Adenosine/AMP deaminase active site	2	2	0.171	0.007
IPR007642	RNA polymerase Rpb2, domain 2	2	2	0.171	0.007
IPR013806	Kringle-like fold	2	2	0.171	0.007
IPR013838	Beta tubulin, autoregulation binding site	2	2	0.171	0.007
IPR016034	Phosphatidylinositol-4-phosphate 5-kinase, core, subgroup	2	2	0.171	0.007
IPR017448	Speract/scavenger receptor-related	2	2	0.171	0.007
IPR023795	Protease inhibitor I4, serpin, conserved site	2	2	0.171	0.007
IPR023796	Serpin domain	2	2	0.171	0.007
00590	Arachidonic acid metabolism	3	2	0.035	0.001
00480	Glutathione metabolism	13	2	0.155	0.014
00564	Glycerophospholipid metabolism	22	2	0.267	0.038
00980	Metabolism of xenobiotics by cytochrome P450	5	2	0.059	0.002

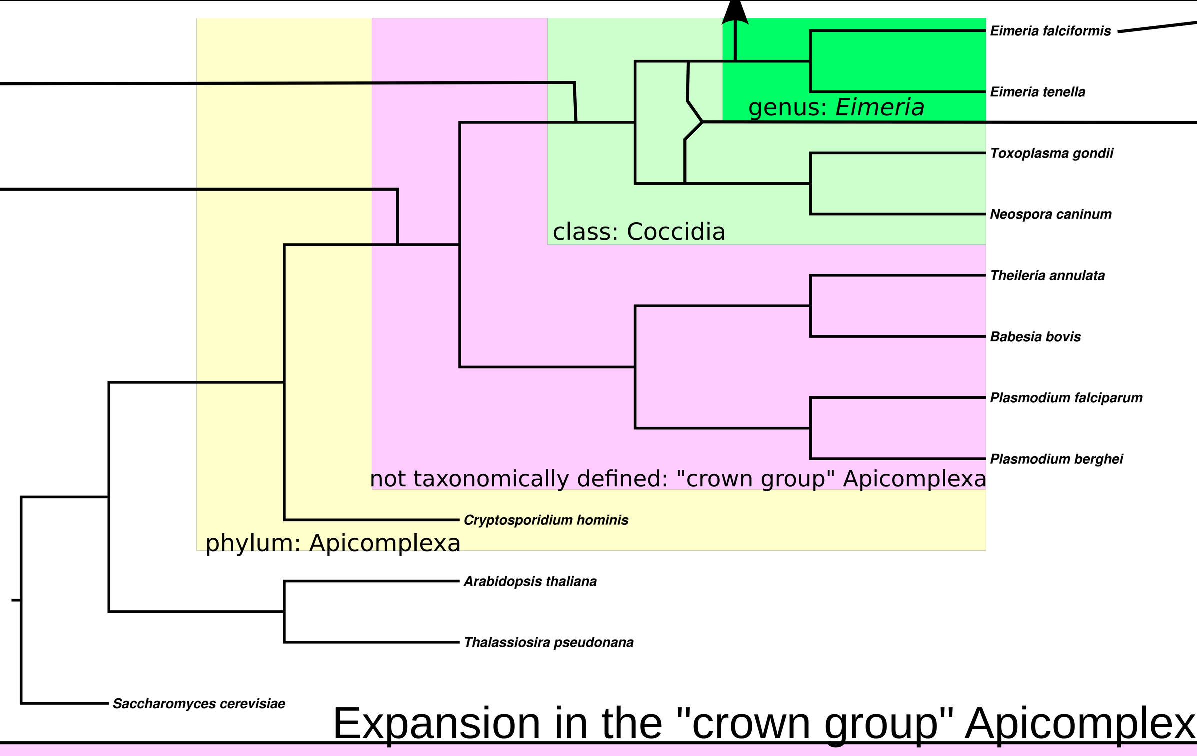

Expansion in the class Coccidia

Id	description	Annotated	In group	Expected	p-value
GO:0003774	motor activity	31	20	2.31	2.9e-16
GO:0003777	microtubule motor activity	22	13	1.64	3.4e-10
GO:0016787	hydrolase activity	413	61	30.78	1.4e-09
GO:0016817	hydrolase activity, acting on acid anhydrides	222	41	16.55	3.5e-09
GO:0017111	nucleoside-triphosphatase activity	215	40	16.02	4.7e-09
GO:0016462	pyrophosphatase activity	218	40	16.25	7.3e-09
GO:0016818	hydrolase activity, acting on phosphorus-containing anhydrides	219	40	16.32	8.4e-09
GO:0008237	metallopeptidase activity	23	10	1.71	1.9e-06
GO:0008061	chitin binding	4	4	0.30	3.0e-05
GO:0097367	carbohydrate derivative binding	4	4	0.30	3.0e-05
GO:0007017	microtubule-based process	34	18	2.54	8.6e-13
GO:0006928	cellular component movement	27	16	2.02	1.8e-12
GO:0007018	microtubule-based movement	27	16	2.02	1.8e-12
GO:0006022	aminoglycan metabolic process	4	4	0.30	2.9e-05
GO:0006030	chitin metabolic process	4	4	0.30	2.9e-05
GO:0006040	amino sugar metabolic process	5	4	0.37	0.00014
GO:1901071	glucosamine-containing compound metabolic process	5	4	0.37	0.00014
GO:0006461	protein complex assembly	10	4	0.75	0.00435
GO:0070271	protein complex biogenesis	10	4	0.75	0.00435
GO:0007596	blood coagulation	2	2	0.15	0.00554
IPR004273	Dynein heavy chain	10	10	0.814	1.4e-11
IPR013602	Dynein heavy chain, domain-2	10	10	0.814	1.4e-11
IPR024743	Dynein heavy chain, coiled coil stalk	10	10	0.814	1.4e-11
IPR024317	Dynein heavy chain, P-loop containing D4 domain	9	9	0.734	1.7e-10
IPR011704	ATPase, dynein-related, AAA domain	8	7	0.655	1.9e-07
IPR001609	Myosin head, motor domain	9	7	0.737	7.9e-07
IPR012779	Peptidase M1, alanyl aminopeptidase	5	5	0.411	3.8e-06
IPR013594	Dynein heavy chain, domain-1	5	5	0.411	3.8e-06
IPR014782	Peptidase M1, membrane alanine aminopeptidase, N-terminal	5	5	0.411	3.8e-06
IPR024601	Peptidase M1, alanyl aminopeptidase, C-terminal	5	5	0.411	3.8e-06
IPR002557	Chitin binding domain	4	4	0.329	4.6e-05
IPR009081	Acyl carrier protein-like	4	4	0.329	4.6e-05
IPR003593	AAA+ ATPase domain	64	15	5.207	1.7e-04
IPR014031	Beta-ketoacyl synthase, C-terminal	5	4	0.411	2.1e-04
IPR006162	Phosphopantetheine attachment site	3	3	0.247	5.6e-04
IPR013120	Male sterility, NAD-binding	3	3	0.247	5.6e-04
IPR013578	Peptidase M16C associated	3	3	0.247	5.6e-04
IPR020806	Polyketide synthase, phosphopantetheine-binding domain	3	3	0.247	5.6e-04
IPR007863	Peptidase M16, C-terminal	10	5	0.822	6.7e-04
IPR011249	Metalloenzyme, LuxS/M16 peptidase-like, metal-binding	10	5	0.822	6.7e-04
IPR001227	Acyl transferase domain	4	3	0.330	0.002
IPR004043	LCCL	4	3	0.330	0.002
IPR014030	Beta-ketoacyl synthase, N-terminal	4	3	0.330	0.002
IPR014043	Acyl transferase	4	3	0.330	0.002
IPR016036	Malonyl-CoA ACP transacylase, ACP-binding	4	3	0.330	0.002
IPR018316	Tubulin/FtsZ, 2-layer sandwich domain	4	3	0.330	0.002
IPR020801	Polyketide synthase, acyl transferase domain	4	3	0.330	0.002
IPR020841	Polyketide synthase, beta-ketoacyl synthase domain	4	3	0.330	0.002
IPR003439	ABC transporter-like	18	6	1.478	0.003
IPR011237	Peptidase M16, core	8	4	0.658	0.003
IPR016035	Acyl transferase/acyl hydrolase/lysophospholipase	8	4	0.658	0.003
IPR016038	Thiolase-like, subgroup	8	4	0.658	0.003
IPR016039	Thiolase-like	8	4	0.658	0.003
IPR017871	ABC transporter, conserved site	14	5	1.151	0.004
IPR001140	ABC transporter, transmembrane domain	9	4	0.741	0.004
IPR011527	ABC transporter, transmembrane domain, type 1	9	4	0.741	0.004
IPR017940	ABC transporter, integral membrane type 1	9	4	0.741	0.004
IPR008280	Tubulin/FtsZ, C-terminal	5	3	0.412	0.004
IPR017975	Tubulin, conserved site	5	3	0.412	0.004
IPR020842	Polyketide synthase/Fatty acid synthase, KR	5	3	0.412	0.004
IPR023123	Tubulin, C-terminal	5	3	0.412	0.004
IPR001757	ATPase, P-type, K/Mg/Cd/Cu/Zn/Na/Ca/Na/H-transporter	10	4	0.823	0.006
IPR023299	ATPase, P-type, cytoplasmic domain N	10	4	0.823	0.006
IPR001190	Speract/scavenger receptor	2	2	0.165	0.006
IPR002453	Beta tubulin	2	2	0.165	0.006
IPR006685	Mechanosensitive ion channel MscS	2	2	0.165	0.006
IPR007621	TPM domain	2	2	0.165	0.006
IPR013838	Beta tubulin, autoregulation binding site	2	2	0.165	0.006
IPR013968	Polyketide synthase, KR	2	2	0.165	0.006
IPR017448	Speract/scavenger receptor-related	2	2	0.165	0.006
IPR020807	Polyketide synthase, dehydratase domain	2	2	0.165	0.006
IPR000217	Tubulin	6	3	0.494	0.009
IPR003008	Tubulin/FtsZ, GTPase domain	6	3	0.494	0.009
IPR008984	SMAD/FHA domain	6	3	0.494	0.009
IPR009030	Growth factor, receptor	11	4	0.905	0.009
IPR018303	ATPase, P-type phosphorylation site	11	4	0.905	0.009
00625	Chloroalkane and chloroalkene degradation	3	2	0.247	0.020
00982	Drug metabolism - cytochrome P450	3	2	0.247	0.020
00565	Ether lipid metabolism	6	3	0.488	0.010
00010	Glycolysis / Gluconeogenesis	29	6	2.309	0.030
00680	Methane metabolism	16	4	1.297	0.040
00626	Naphthalene degradation	2	2	0.165	0.007
00830	Retinol metabolism	2	2	0.165	0.007

Independent expansion in *Eimeria* and Sarcocystidae

Id	description	Annotated	In group	Expected	p-value
GO:0008237	metallopeptidase activity	23	5	0.23	1.8e-06
GO:0004332	fructose-bisphosphate aldolase activity	2	2	0.02	9.8e-05
GO:0016832	aldehyde-lyase activity	2	2	0.02	9.8e-05
GO:0016787	hydrolase activity	413	12	4.20	0.00010
GO:0042626	ATPase activity, coupled to transmembrane movement of substances	31	4	0.32	0.00020
GO:0043492	ATPase activity, coupled to movement of substances	31	4	0.32	0.00020
GO:0016820	hydrolase activity, catalyzing transmembrane movement of substances	33	4	0.34	0.00025
GO:0015399	primary active transmembrane transporter activity	35	4	0.36	0.00032
GO:0015405	P-P-bond-hydrolysis-driven transmembrane transporter activity	35	4	0.36	0.00032
GO:0022857	transmembrane transporter activity	70	5	0.71	0.00048
GO:0051179	localization	184	7	2.12	0.0022
GO:0055085	transmembrane transport	66	4	0.76	0.0053
GO:0006096	glycolysis	12	2	0.14	0.0076
GO:0006007	glucose catabolic process	16	2	0.18	0.0135
GO:0016052	carbohydrate catabolic process	16	2	0.18	0.0135
GO:0019320	hexose catabolic process	16	2	0.18	0.0135
GO:0044724	single-organism carbohydrate catabolic process	16	2	0.18	0.0135
GO:0046365	monosaccharide catabolic process	16	2	0.18	0.0135
GO:0044699	single-organism process	332	8	3.82	0.0173
GO:0006006	glucose metabolic process	21	2	0.24	0.0228
IPR012779	Peptidase M1, alanyl aminopeptidase	5	5	0.043	6.0e-11
IPR014782	Peptidase M1, membrane alanine aminopeptidase, N-terminal	5	5	0.043	6.0e-11
IPR024601	Peptidase M1, alanyl aminopeptidase, C-terminal	5	5	0.043	6.0e-11
IPR001140	ABC transporter, transmembrane domain	9	4	0.079	8.3e-07
IPR011527	ABC transporter, transmembrane domain, type 1	9	4	0.079	8.3e-07
IPR017940	ABC transporter, integral membrane type 1	9	4	0.079	8.3e-07
IPR017871	ABC transporter, conserved site	14	4	0.123	6.4e-06
IPR003439	ABC transporter-like	18	4	0.158	1.9e-05
IPR000741	Fructose-bisphosphate aldolase, class-I	2	2	0.018	8.5e-05
IPR009543	Vacuolar protein sorting-associated protein	4	2	0.036	0.001
IPR023174	3'5'-cyclic nucleotide phosphodiesterase, conserved site	5	2	0.045	0.001
IPR002073	3'5'-cyclic nucleotide phosphodiesterase, catalytic domain	6	2	0.054	0.001
IPR023088	3'5'-cyclic nucleotide phosphodiesterase	6	2	0.054	0.001
IPR003593	AAA+ ATPase domain	64	4	0.565	0.003
IPR002259	Equilibrative nucleoside transporter	1	1	0.009	0.009
IPR008636	HOOK	1	1	0.009	0.009
IPR013534	Starch synthase, catalytic domain	1	1	0.009	0.009
IPR013785	Aldolase-type TIM barrel	17	2	0.154	0.011
IPR000177	Apple domain	2	1	0.018	0.019
IPR003014	PAN-1 domain	3	1	0.028	0.028
IPR003609	Apple-like	3	1	0.028	0.028
IPR021691	Protein of unknown function DUF3273	3	1	0.028	0.028
00710	Carbon fixation in photosynthetic organisms	14	2	0.272	0.035
00051	Fructose and mannose metabolism	11	2	0.213	0.022
00480	Glutathione metabolism	13	2	0.253	0.031
00680	Methane metabolism	16	2	0.312	0.045
00030	Pentose phosphate pathway	15	2	0.292	0.040

Expansion in the "crown group" Apicomplexa

Id	description	Annotated	In group	Expected	p-value
GO:0003735	structural constituent of ribosome	95	43	21.62	5.0e-07
GO:0005198	structural molecule activity	111	46	25.26	4.2e-06
GO:0003774	motor activity	31	17	7.05	9.3e-05
GO:0003899	DNA-directed RNA polymerase activity	23	13	5.23	0.00043
GO:0034062	RNA polymerase activity	23	13	5.23	0.00043
GO:0008061	chitin binding	4	4	0.91	0.00265
GO:0019207	kinase regulator activity	4	4	0.91	0.00265
GO:0019887	protein kinase regulator activity	4	4	0.91	0.00265
GO:0097367	carbohydrate derivative binding	4	4	0.91	0.00265
GO:0016779	nucleotidyltransferase activity	42	18	9.56	0.00267
GO:0006412	translation	158	61	38.59	1.5e-05
GO:0009059	macromolecule biosynthetic process	242	85	59.11	1.8e-05
GO:0034645	cellular macromolecule biosynthetic process	242	85	59.11	1.8e-05
GO:0010467	gene expression	265	88	64.72	0.00016
GO:0009058	biosynthetic process	348	110	85.00	0.00018
GO:1901576	organic substance biosynthetic process	334	106	81.58	0.00021
GO:0044267	cellular protein metabolic process	355	111	86.71	0.00029
GO:0044249	cellular biosynthetic process	334	105	81.58	0.00037
GO:0009987	cellular process	937	249	228.86	0.00070
GO:0019538	protein metabolic process	422	126	103.07	0.00091
IPR004273	Dynein heavy chain	10	10	2.234	3.1e-07
IPR013602	Dynein heavy chain, domain-2	10	10	2.234	3.1e-07
IPR024743	Dynein heavy chain, coiled coil stalk	10	10	2.234	3.1e-07
IPR024317	Dynein heavy chain, P-loop containing D4 domain	9	9	2.011	1.4e-06
IPR011704	ATPase, dynein-related, AAA domain	8	7	1.790	1.8e-04
IPR011332	Ribosomal protein, zinc-binding domain	5	5	1.120	0.001
IPR012779	Peptidase M1, alanyl aminopeptidase	5	5	1.120	0.001
IPR013594	Dynein heavy chain, domain-1	5	5	1.120	0.001
IPR014782	Peptidase M1, membrane alanine aminopeptidase, N-terminal	5	5	1.120	0.001
IPR024601	Peptidase M1, alanyl aminopeptidase, C-terminal	5	5	1.120	0.001
IPR001609	Myosin head, motor domain	9	7	2.014	0.001
IPR016024	Armadillo-type fold	72	5	16.278	0.001
IPR010920	Like-Sm (LSM) domain	14	9	3.131	0.001
IPR011989	Armadillo-like helical	58	4	13.095	0.002
IPR000555	JAB1/Mov34/MPN/PAD-1	4	4	0.896	0.003
IPR002557	Chitin binding domain	4	4	0.896	0.003
IPR001163	Ribonucleoprotein LSM domain	11	7	2.462	0.004
IPR023313	Ubiquitin-conjugating enzyme, active site	11	7	2.462	0.004
IPR000608	Ubiquitin-conjugating enzyme, E2	12	7	2.686	0.008
00190	Oxidative phosphorylation	33	12	6.864	0.048
00440	Phosphonate and phosphinate metabolism	4	4	0.844	0.002
00230	Purine metabolism	61	20	12.475	0.033
00240	Pyrimidine metabolism	52	21	10.400	0.001
